# Glaucoma is associated with plasmin proteolytic activation mediated through oxidative inactivation of neuroserpin

**DOI:** 10.1038/s41598-017-08688-2

**Published:** 2017-08-21

**Authors:** Vivek Gupta, Mehdi Mirzaei, Veer Bala Gupta, Nitin Chitranshi, Yogita Dheer, Roshana Vander Wall, Mojdeh Abbasi, Yuyi You, Roger Chung, Stuart Graham

**Affiliations:** 1 Faculty of Medicine and Health Sciences, Macquarie University, Sydney, Australia; 2 Department of Chemistry and Biomolecular Sciences, Macquarie University, Sydney, Australia; 3 School of Medical Sciences, Edith Cowan University, Perth, Australia; 4 Save Sight Institute, Sydney University, Sydney, Australia

## Abstract

Neuroserpin is a serine protease inhibitor that regulates the activity of plasmin and its activators in the neuronal tissues. This study provides novel evidence of regulatory effect of the neuroserpin on plasmin proteolytic activity in the retina in glaucoma. Human retinal and vitreous tissues from control and glaucoma subjects as well as retinas from experimental glaucoma rats were analysed to establish changes in plasmin and neuroserpin activity. Neuroserpin undergoes oxidative inactivation in glaucoma which leads to augmentation of plasmin activity. Neuroserpin contains several methionine residues in addition to a conserved reactive site methionine and our study revealed enhanced oxidation of Met residues in the serpin under glaucoma conditions. Met oxidation was associated with loss of neuroserpin inhibitory activity and similar findings were observed in the retinas of superoxide dismutase (SOD) mutant mice that have increased oxidative stress. Treatment of purified neuroserpin with H2O2 further established that Met oxidation inversely correlated with its plasmin inhibitory activity. Dysregulation of the plasmin proteolytic system associated with increased degradation of the extracellular matrix (ECM) proteins in the retina. Collectively, these findings delineate a novel molecular basis of plasmin activation in glaucoma and potentially for other neuronal disorders with implications in disease associated ECM remodelling.

## Introduction

Glaucoma is the most common cause of irreversible vision loss marked by retinal ganglion cell (RGC) degeneration and excavation of the optic nerve head. Increased intraocular pressure (IOP) is a prominent manifestation of glaucoma and controlling IOP remains the primary means of disease management. Several factors such as pressure induced remodelling of the lamina cribrosa, axonal compression of the RGCs, obstruction in the retrograde flow of neurotrophins to RGCs, impediments in axonal transport along the optic nerve, chronic ischemic insult and digestion of the extracellular matrix (ECM) by proteolytic activity have been suggested to play a role in the glaucoma pathology^1–3^. The primary site of glaucoma induced damage is also debatable with some studies suggesting RGC damage occurring first followed by optic nerve head (ONH) excavation while others suggesting the reverse of this process^4^. Regardless, there is a need to better understand the molecular basis of RGC loss and optic nerve excavation. Involvement of proteases, particularly the proteolytic activity of serine protease plasmin is implicated in inducing excitotoxic damage to retina and RGC in glaucoma^5–7^. Various studies suggest that plasmin activation promotes microglial activation and compromises blood brain barrier (BBB)^8^. In the eye, the enzyme plays a role in maintaining the normal retinal integrity and there is evidence to suggest that inhibition of plasmin or plasminogen activators attenuates the death of RGCs *in vivo* as well as *in vitro* ^5, 9^. The extent of plasmin involvement in glaucoma pathology and mechanisms underlying its regulation in retina however, remain unclear.

The serine protease inhibitor, neuroserpin is shown to inhibit both plasmin and its activators, tissue plasminogen activator (tPA) and urokinase-type plasminogen activator (uPA) in the neuronal tissues^10^. The serpin was initially identified as an axonally released protein from dorsal root ganglion neurons in chicken and plays an important part in neuronal plasticity and survival, synaptic network formation and in brain development^11–13^. Neuroserpin genetic alterations are linked with familial encephalopathy with neuroserpin inclusion bodies (FENIB) leading to endoplasmic reticulum (ER) retention of the protein and neuronal loss^14^. In addition to the CNS, an increased expression of neuroserpin has been identified in prostate cancer and the molecule is shown to play a role in suppressing the growth of brain tumours^15, 16^. Metastatic lung and breast cancer cells in brain depict increased neuroserpin expression and this is shown to suppress the negative effects of plasmin activation^17^.

Neuroserpin exerts protective effects in neurons against excitotoxicity both *in vitro* and *in vivo* ^18^. Its proteolytic inhibitory activity is shown to protect neurons against damage caused by plasmin activation^19^. Mice overexpressing neuroserpin exhibited loss of tPA activity in the brain^20^. Neuroserpin overexpression or deficiency of plasmin family members is shown to correlate with protective outcomes in ischemic stroke^21^. In the visual cortex neuroserpin expression is upregulated during development^22^. Recent studies indicate that neuroserpin administration suppresses apoptotic pathways in RGCs in response to acute retinal ischemic injury and protect against loss of retinal function^23^.

To investigate whether plasmin and its endogenous antagonist neuroserpin are affected in glaucoma, this study for the first time evaluated the expression, activity, localisation and protein-protein interaction changes of the two proteins in human post-mortem samples as well as in a rat model of experimental glaucoma. The results provide novel data on the plasmin and neuroserpin changes under pathological conditions as well as offer mechanistic insights into the neuroserpin mediated regulation of plasmin activity. Oxidative inactivation of neuroserpin was identified as the molecular basis of increased plasmin activity in glaucoma conditions. Biochemical investigations were compared between the human glaucoma samples and the animal model of experimental glaucoma. Further, the impact of oxidative stress on the plasmin-neuroserpin system was corroborated using an *in vitro* model as well as in the superoxide dismutase mutant mice. The findings are expected to open up avenues to use plasmin specific inhibitors as a mechanism based strategy in glaucoma treatment. The focus of the study was primarily to advance knowledge of the mechanism underlying glaucoma pathogenesis but may have relevance to other health and neurodegenerative conditions linked with oxidative stress and imbalance of plasmin-neuroserpin equilibrium.

## Results

### Neuroserpin and plasmin expression and glaucoma associated changes in the human retinal and vitreous tissues

**Figure 1:**
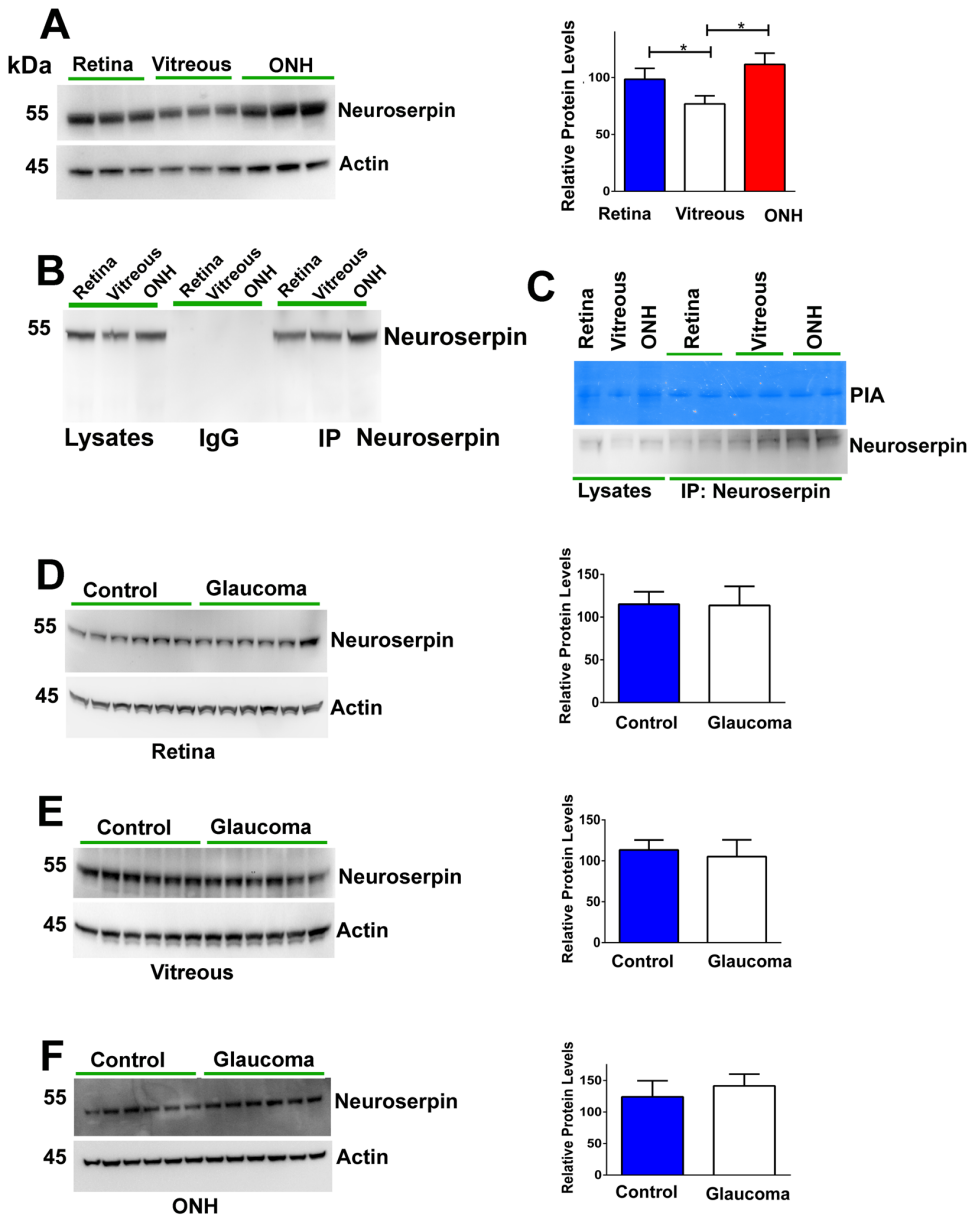
(**A**) Western blot showing neuroserpin expression in the human retina, vitreous and ONH and densitometric quantification of the neuroserpin band intensity (n = 6; *p < 0.05). (**B**) Neuroserpin was immunoprecipitated from human retina, vitreous and ONH samples using anti-neuroserpin antibody (SC32947) and blots probed for neuroserpin immunoreactivity using another neuroserpin antibody (SC48360). Non-reactive IgG was used as control in each case along with corresponding tissue lysates. (**C**) Plasmin inhibitory activity of neuroserpin immunoprecipitated from human retina, vitreous and ONH was assessed using gelatin gel zymography. (**D–F**) Human retina, vitreous and ONH tissue lysates were immunoblotted and probed with anti-neuroserpin antibody using samples obtained from both control and glaucoma subjects (n = 12). Blots were cropped to show the relevant band. The relative intensities of bands in WB were quantified and plotted. Actin was used as internal loading control in each WB.

We investigated the neuroserpin expression in the healthy human retina, ONH region and in the vitreous using western blotting (WB). Neuroserpin was well expressed in the retina, ONH and vitreous tissues. Densitometric quantification of the bands indicated that the neuroserpin expression was significantly higher in the retina and ONH compared to the vitreous (p < 0.05) (Fig. 1A). Neuroserpin immunoprecipitation followed by probing with a different neuroserpin antibody established that anti-neuroserpin antibody could be effectively used to pull down the protein from the retinal and vitreous tissues. Non-immune immunoglobulin was used as control for immunoprecipitation (Fig. 1B). Primary structural alignment also showed high sequence similarity between human, rat and mouse neuroserpin (Fig. S2). The plasmin protease inhibitory activity of the neuroserpin in tissue lysates and immunoprecipitates was tested using in-gel gelatin zymography revealing protease inhibitory activity of neuroserpin in each case (Fig. 1C). To investigate whether neuroserpin levels exhibit any alterations under glaucoma conditions, retinal, ONH and vitreous tissue lysates from human control and glaucoma samples were subjected to immunoblotting analysis using actin as a loading control. Densitometric evaluation of band intensities indicate that neuroserpin expression was not altered in either of these glaucoma tissues compared to controls (Fig. 1D–F).

**Figure 2:**
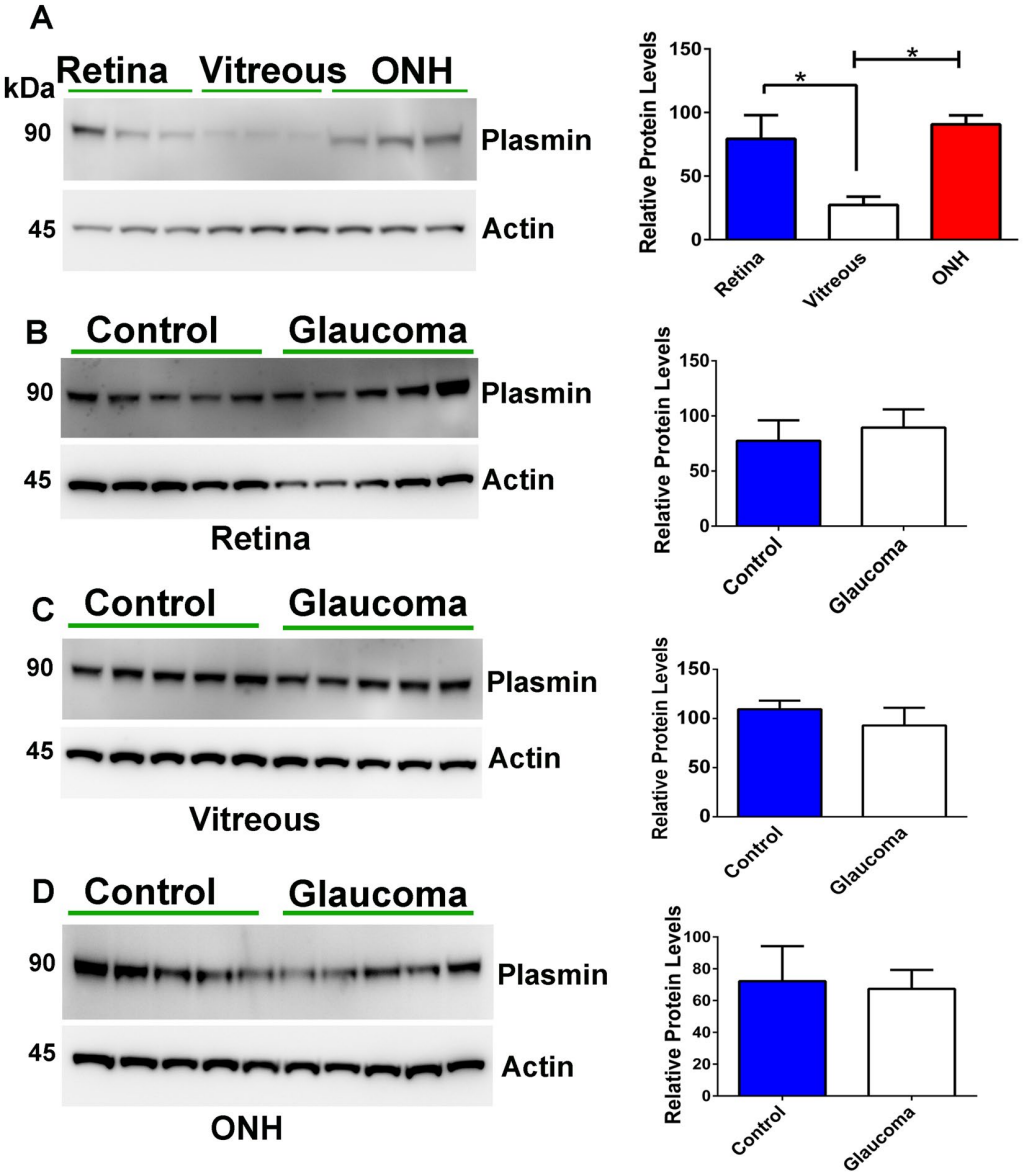
(**A**) Plasmin expression was evaluated in the human retina, vitreous and ONH using immunoblotting and relative band intensities quantified (n = 6; *p < 0.01). (**B–D**) Plasmin expression levels were also evaluated in the human retinal, vitreous and ONH samples from glaucoma and control subjects using western blotting and relative band intensities quantified and plotted (n = 5 each). Blots were cropped to show the relevant band. Actin was used for loading normalisation in each case.

Plasmin expression in the normal human vitreous, retina and ONH samples was also studied using WB and results show that the enzyme was well expressed in all these tissues. Retina and ONH demonstrate significantly higher expression of plasmin compared to vitreous using actin as the loading control (p < 0.01) (Fig. 2A). To determine potential alterations in the plasmin protein expression under glaucoma conditions, we also tested the retinal, vitreous and ONH lysates from control and glaucoma subjects. WB densitometric quantification demonstrated that plasmin expression was not significantly altered under glaucoma conditions (Fig. 2B–D).

### Neuroserpin and plasmin expression and localisation in experimental model of increased IOP

**Figure 3:**
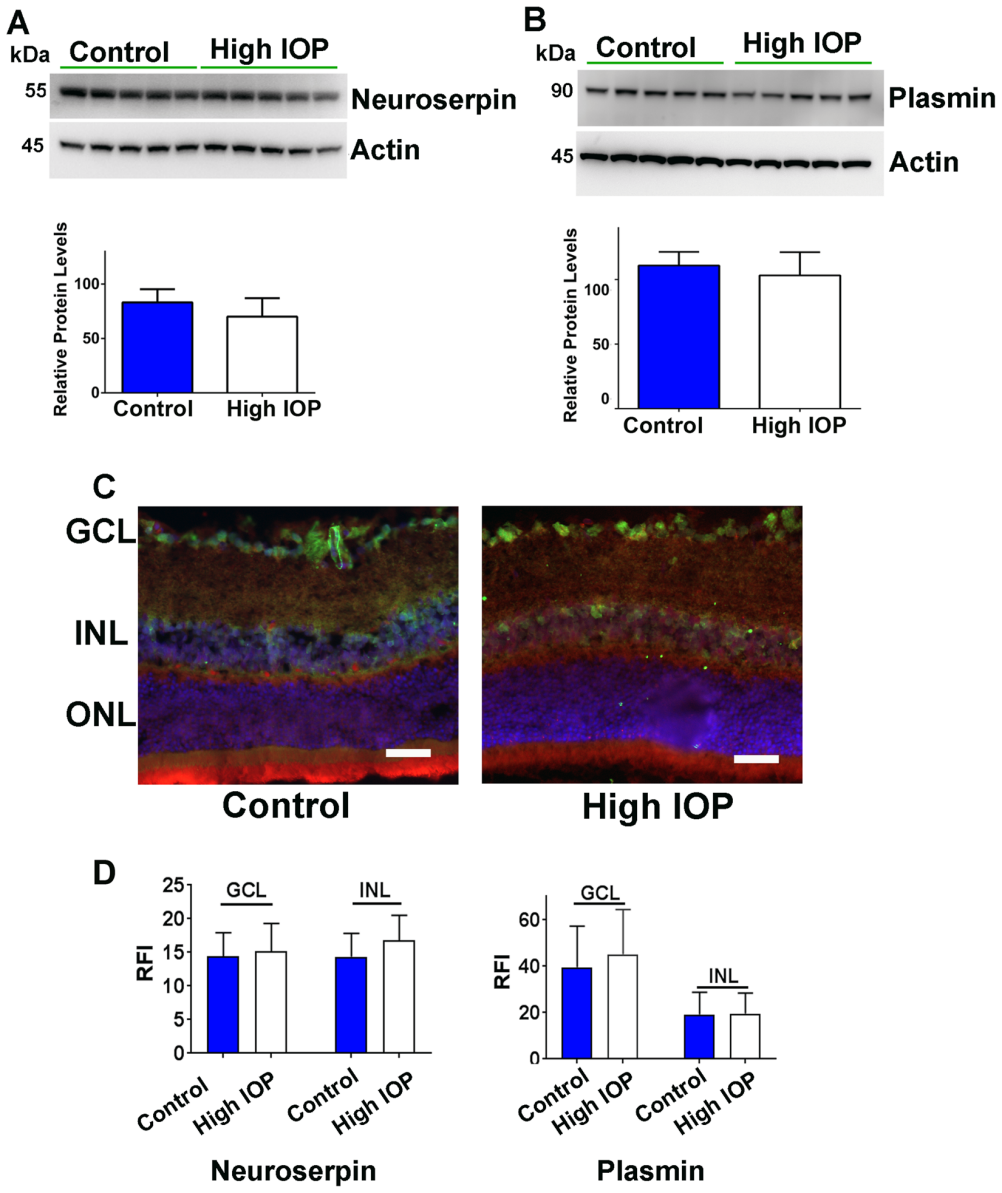
Rat retinal lysates from control and high IOP rats were subjected to western blotting and probed for (**A**) neuroserpin and (**B**) plasmin immunoreactivity. Actin was used as loading control. Blots were cropped to show the relevant band. The relative intensities of WB bands were quantified in both cases and plotted (n = 5 each). (**C**) Double immunostaining of the rat retinal sections from control and microbead induced high IOP eyes illustrating neuroserpin (red) and plasminogen (green) expression and localization in various retinal layers (Blue-DAPI). (n = 5; scale 50 µm). (**D**) Relative fluorescence intensities of neuroserpin and plasmin immunoreactivity in sections were quantified using ImageJ programme in GCL and INL and plotted.

The impact of chronically elevated IOP on the neuroserpin and plasmin protein expression and localisation in the retina was tested in an animal model of glaucoma. The rat model of experimental glaucoma using microbead injections resulted in chronically elevated IOP with a sustained increase from 10.8 ± 1.3 to 21.6 ± 1.5 mmHg in these animals for a period of 2 months (Fig. S1). At the end-point, rat retinal tissues were harvested and analysed for neuroserpin and plasmin protein expression changes using WB and immunofluorescence. Similar to the human glaucoma results, the experimental glaucoma model also did not indicate any IOP induced changes in neuroserpin or plasmin expression (Fig. 3A,B). Further, the protein localisation was evaluated in the rat retinal sections using immunofluorescence staining. Results suggested that both neuroserpin (red) and plasmin (green) expression was largely consistent under the control and glaucoma conditions (Fig. 3C). Densitometric quantification of the relative fluorescence intensity (RFI) revealed no significant changes in the expression of neuroserpin or plasmin within the GCL or inner nuclear layer (INL) (Fig. 3D). Together, these results indicate that neuroserpin and plasmin expression and their localisation within various retinal layers is not altered under glaucomatous conditions.

### Enhanced plasmin proteolytic activity in retinal and vitreous tissues in glaucoma

**Figure 4:**
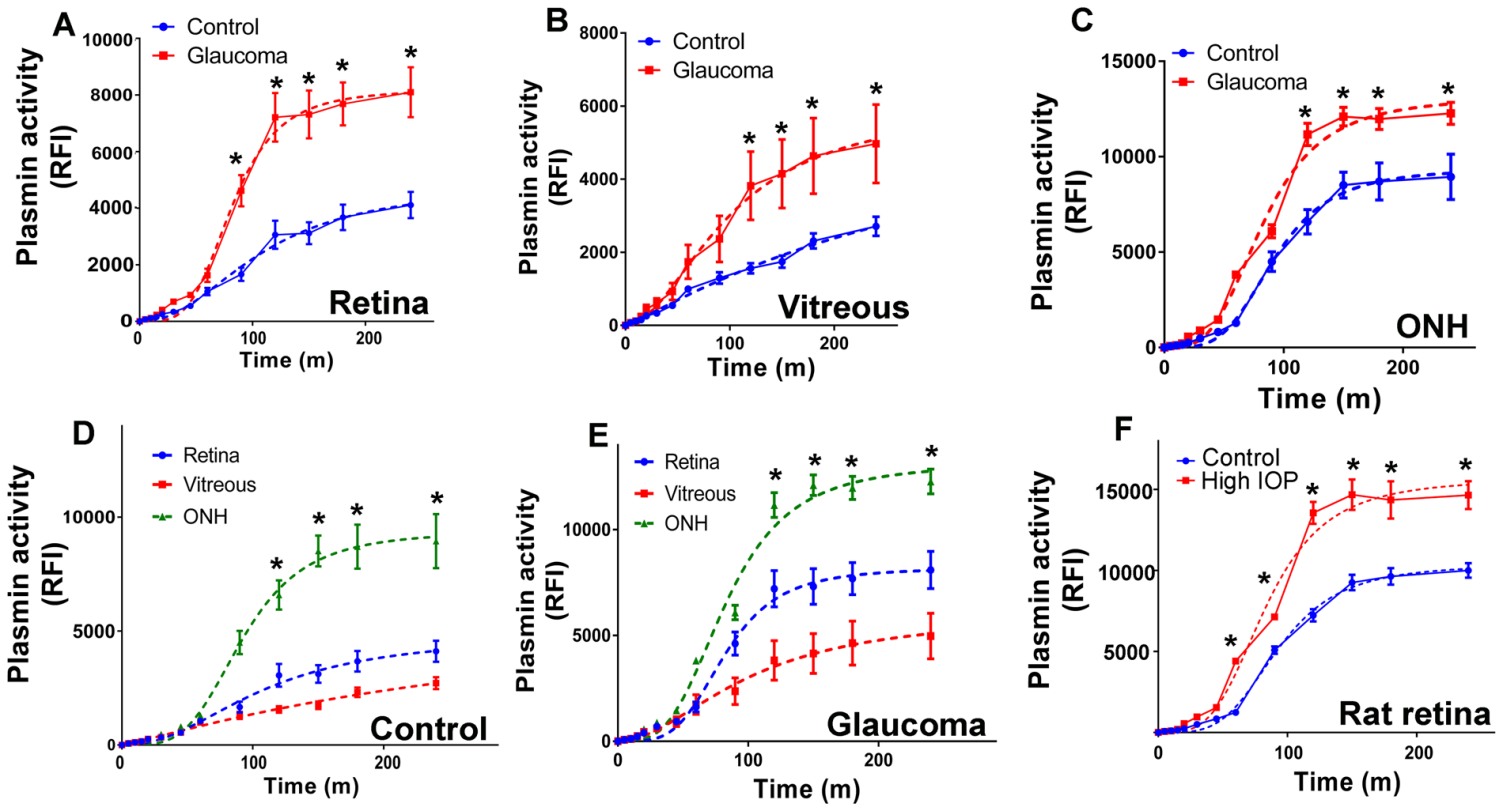
Plasmin proteolytic activity was measured in tissue lysates from (**A**) human retina(**B**) vitreous and (**C**) ONH samples from both control (n = 12) and glaucoma eyes (n = 12) in a time dependent manner (0–300 minutes) and data plotted. Data was fitted using allosteric sigmoidal curve and regression analysis carried out. (**D–E**) Plasmin proteolytic activity change in retinal, vitreous and ONH tissues from control and glaucoma samples using data from figures A–C is re-plotted together for comparison. (**F**) Time dependent plasmin proteolytic activity changes were measured in rat retinal lysates (0–300 minutes) from control (n = 5) and high IOP (n = 5) eyes and data plotted. Dotted lines represent sigmoidal allosteric regression analysis (*p < 0.05).

We further investigated the protease activity of plasmin separately in the human retinal, vitreous and ONH tissue lysates under both normal and glaucoma conditions. Continuous assay of plasmin activity was carried out utilising a Sensolyte Rh110 fluorogenic peptide substrate assay kit^24, 25^ (Anaspec, USA) as per manufacturer’s instructions. Briefly, 30 µg of tissue extract was incubated with 50 µL of fluorimetric substrate and Ex/Em: 490/520 nm recorded in triplicates. Relative fluorescent intensity (RFI) changes were assessed in a time dependent manner from 0–300 minutes at indicated time points and data compared to control values. Results indicate that glaucoma samples depicted a significantly elevated plasmin amidolytic activity in glaucoma compared to controls (Fig. 4A–C). Enzymatic activity plots demonstrated a multiphasic sigmoidal slope (dotted line) with an initial log phase that eventually reached a plateau (R^2^: retina control = 0.96; retina glaucoma = 0.97; vitreous control = 0.97; vitreous glaucoma = 0.9; ONH control = 0.97; ONH glaucoma = 0.98). The plasmin enzymatic activity was also compared between retina, vitreous and ONH samples under the control and glaucomatous conditions (Fig. 4D,E). Data analysis showed that in the control samples the proteolytic activity was highest in the ONH followed by whole retinal lysates and vitreous respectively. Similarly, glaucoma samples also depicted highest plasmin activity in ONH followed by retina and vitreous tissues (Fig. 4D–E). We further investigated the impact of chronic exposure of elevated IOP on the plasmin activity in retina (0–300 minutes) in the rat model of experimental glaucoma. In parallel to the observations from human glaucoma samples, significantly elevated plasmin activity was detected in the rat retinas that were subjected to increased IOP compared to the corresponding control eyes. Plasmin activity plots from rat retinas also exhibited a non-linear allosteric sigmoidal curve (R^2^ = control 0.98, glaucoma = 0.96) with an initial log phase followed by a plateau stage (Fig. 4F). Furthermore, protein expression analysis of plasminogen activators in the retina was investigated and analysis revealed that tPA and uPA protein levels were essentially unchanged in glaucoma conditions in the human samples (Figs S3A and S4A). A statistical significant increase in tPA levels was observed in the rat retinas exposed to increased IOP for 2 months (Fig. S3B), however no change in uPA levels was observed in the rat retinas at this time point (Fig. S4B). Enzyme activity assays for both tPA and uPA were also carried out in a time dependent manner from 0–8 hrs and relative absorbance change plotted (Figs S3C and S4C). Results indicate that human glaucoma samples did not have any significant change in either tPA or uPA amidolytic activity compared to the control samples. No statistically significant change in tPA amidolytic activity was also observed in rat retinas (Fig. S3C), however interestingly, a higher uPA amidolytic activity was detected in the rat retinas exposed to high IOP (Fig. S4C). Both tPA and uPA activity plots depicted a multiphasic sigmoidal slope (dotted line) (Figs S3C and S4C) (tPA human: R^2^ = control 0.91; glaucoma = 0.88; uPA human: R^2^ = control 0.93; glaucoma = 0.90) (tPA rat: R^2^ = control 0.66; glaucoma = 0.67; uPA rat: R^2^ = control 0.60; high IOP = 0.81) with an initial log phase followed by a plateau stage.

### Plasmin inhibitory activity of neuroserpin is significantly impaired under glaucomatous conditions

**Figure 5:**
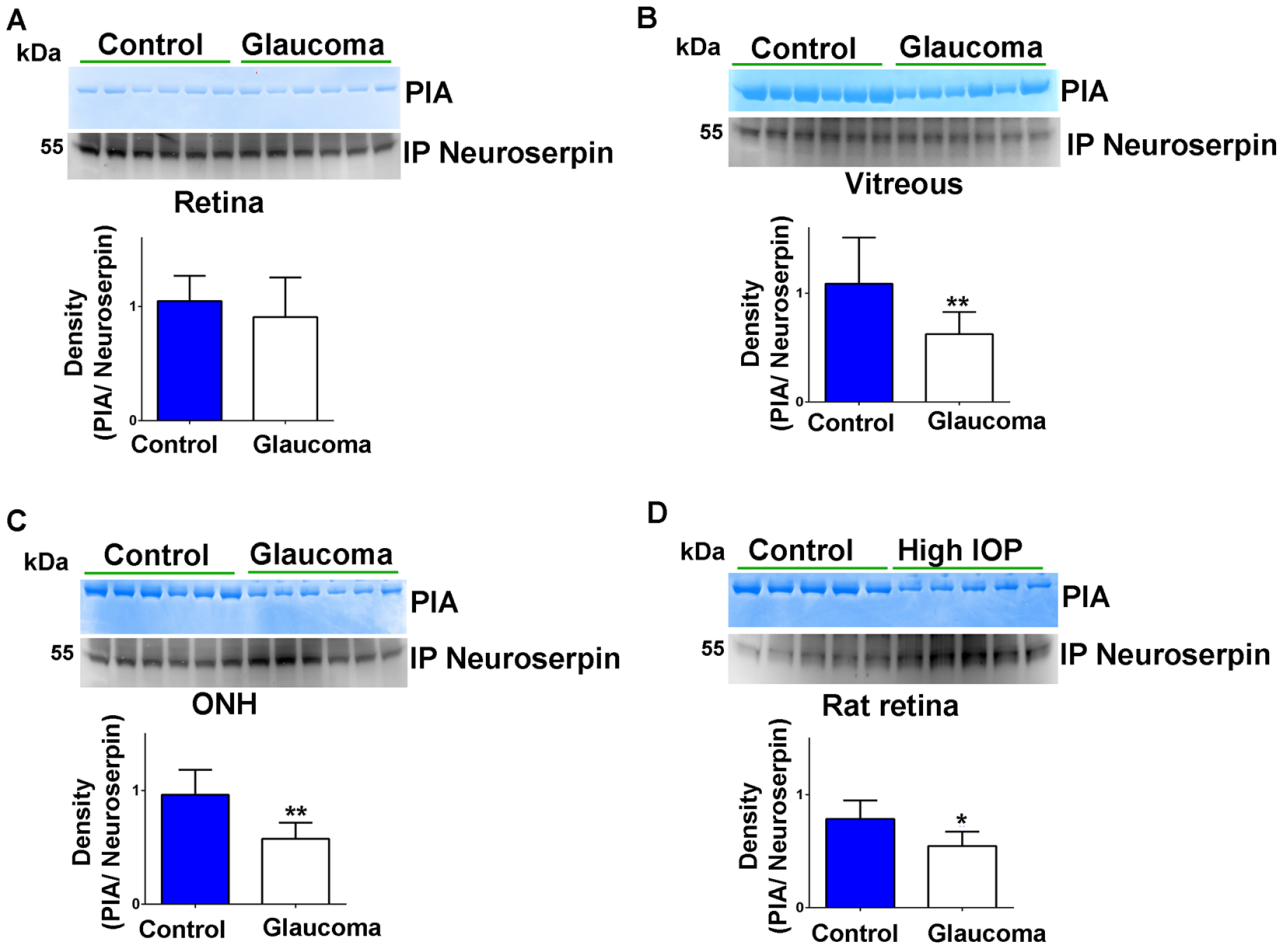
Neuroserpin was immunoprecipitated from (**A**) human retinal (**B**) vitreous and (**C**) ONH tissue lysates using anti-neuroserpin antibody and subjected to gelatin zymography to evaluate the plasmin inhibitory activity of the neuroserpin (n = 6 each). (**D**) Immunoprecipitated neuroserpin from control and high IOP rat retinas were subjected to gelatin gel zymography to assess its plasmin inhibitory activity (n = 5). Immunoprecipitates were also loaded for western blotting and developed for neuroserpin immunoreactivity in each case. Blots were cropped to show the relevant band. Relative band intensities were quantified and data analysis indicated significantly decreased plasmin inhibitory activity in human vitreous and ONH glaucoma samples and high IOP induced rat retinas compared to the respective controls (*p < 0.05; **p < 0.01).

The plasmin inhibitory activity of neuroserpin was assessed using gelatin in-gel zymography in the whole human retinal lysates, vitreous and ONH. This was carried out following neuroserpin immunoprecipitation using specific antibodies and by incubating the gel with plasmin protease (Fig. 5A–C). Densitometric quantification analysis revealed that the plasmin inhibitory capacity of neuroserpin was significantly impaired in the human vitreous (p < 0.05) and ONH samples isolated from glaucoma subjects compared to the controls (p < 0.01) (Fig. 5B,C). The loss of plasmin inhibitory activity in the whole human retinal lysates did not reach statistical significance and this could potentially be caused by non-homogenous nature of the tissue and combined effect of all retinal regions/cells leading to blurring of specific signal. The effects of retinal exposure to chronically increased IOP on the plasmin inhibitory activity of neuroserpin was also evaluated using the rat model of experimental glaucoma. Neuroserpin immunoprecipitates from high IOP exposed animals when subjected to gelatin in-gel zymography depicted significantly reduced plasmin inhibitory activity compared to the control eyes (p < 0.05). These results along with the human ONH and vitreous observations collectively indicate that plasmin inhibitory activity of neuroserpin although not completely lost was significantly compromised in human as well as experimental glaucoma conditions.

### Neuroserpin-plasmin interactions in the retina in glaucoma

**Figure 6:**
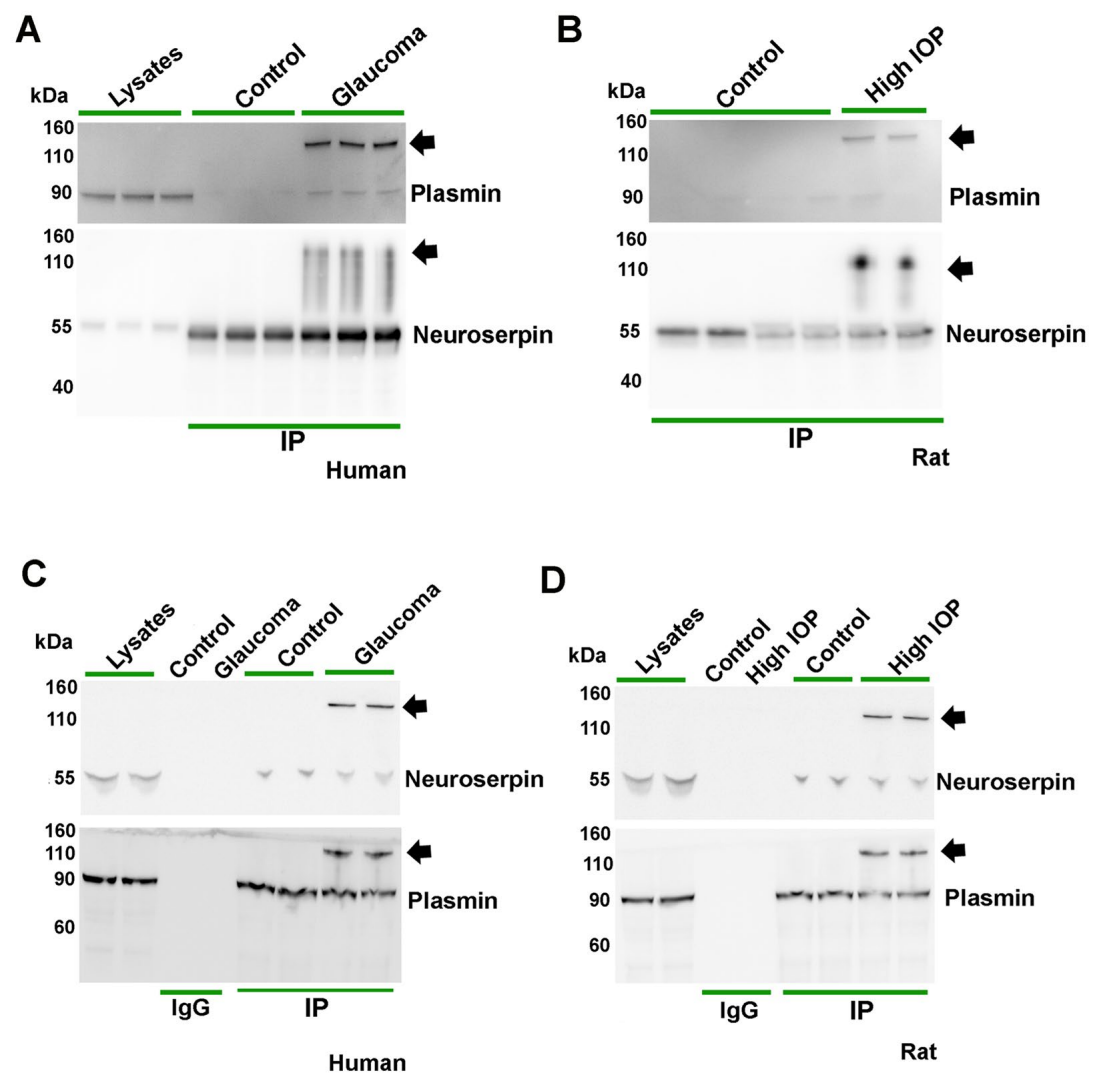
Neuroserpin was immunoprecipitated from (**A**) control and glaucoma human and (**B**) control and high IOP rat retinas. The eluted immunoprecipitates were subjected to western blotting and probed using plasmin and neuroserpin antibodies. Human retinal lysates alone were also loaded as controls. Plasmin was immunoprecipitated from (**C**) human and (**D**) rat retinal lysates from control and glaucoma samples along with corresponding IgG control immunoprecipitations. Human and rat retinal lysates respectively were loaded in C and D. After elution, the immunoprecipitates were subjected to western blotting and probed using plasmin and neuroserpin antibodies. High molecular weight bands (indicated by arrow) implicating neuroserpin-plasmin complex formation were observed in both human and rat glaucomatous retinal samples. Blots were cropped to show the relevant bands.

Human retinal lysates from control and glaucoma samples were incubated with anti-neuroserpin antibody and subjected to immunoprecipitation. The resultant precipitates were eluted from the beads using Gly-HCl, subjected to WB and probed for both neuroserpin and plasmin immunoreactivity. Immunoprecipitation analysis revealed that the plasmin co-immunoprecipitated along with neuroserpin indicating interactions between these two proteins in the retina. We also identified a higher molecular mass band (arrow) indicating complex formation between neuroserpin and plasmin in the retinal immunoprecipitates obtained from glaucoma subjects (Fig. 6A). Human retinal lysates were loaded as controls. The interaction of plasmin with neuroserpin was further confirmed using mass spectrometric (MS) analysis. Following neuroserpin immunoprecipitation and elution of the bound proteins with Gly-HCl buffer, the samples were loaded on to SDS-PAGE, stained with coomassie and lane excised. The proteins were digested and subjected to MS analysis. The peptide sequences obtained were analysed using GenBank and highest number of peptides were identified as belonging to plasminogen (S5). Further, comparing the control and glaucoma samples the relative spectral counts of plasminogen peptides was significantly higher in the glaucoma (14 ± 0.5) compared to control samples (8 ± 1) (p < 0.01). These MS results substantiate the findings from co-immunoprecipitation experiment. The effects of chronically elevated IOP on the plasmin-neuroserpin interaction was also analysed using the rat model of experimental glaucoma. Neuroserpin immunoprecipitation from control and high IOP exposed eyes followed by immunoblot analysis of the eluted proteins revealed faint bands of plasmin as well as neuroserpin-plasmin complex formation (arrow) under high IOP conditions (Fig. 6B). These findings correspond with observations from human samples and indicated enhanced neuroserpin-plasmin complex formation in the retina under glaucomatous conditions. The protein-protein interaction results were further validated by reciprocal immunoprecipitations in both human (Fig. 6C) and rat (Fig. 6D) retinal samples using anti-plasmin antibodies. This experiment revealed that neuroserpin co-immunoprecipitated with the plasmin and a complex formation between plasmin and neuroserpin (arrow) was identified in both human and rat immunoprecipitates under glaucomatous conditions. Retinal lysates alone and immunoprecipitations with non-immune immunoglobulins were used as controls. These results indicated that neuroserpin interacted with plasmin in the retina and complex formation between the two proteins was significantly increased or stabilised under glaucoma conditions.

### Methionine oxidation and inactivation of neuroserpin under glaucoma and oxidative stress conditions

**Figure 7:**
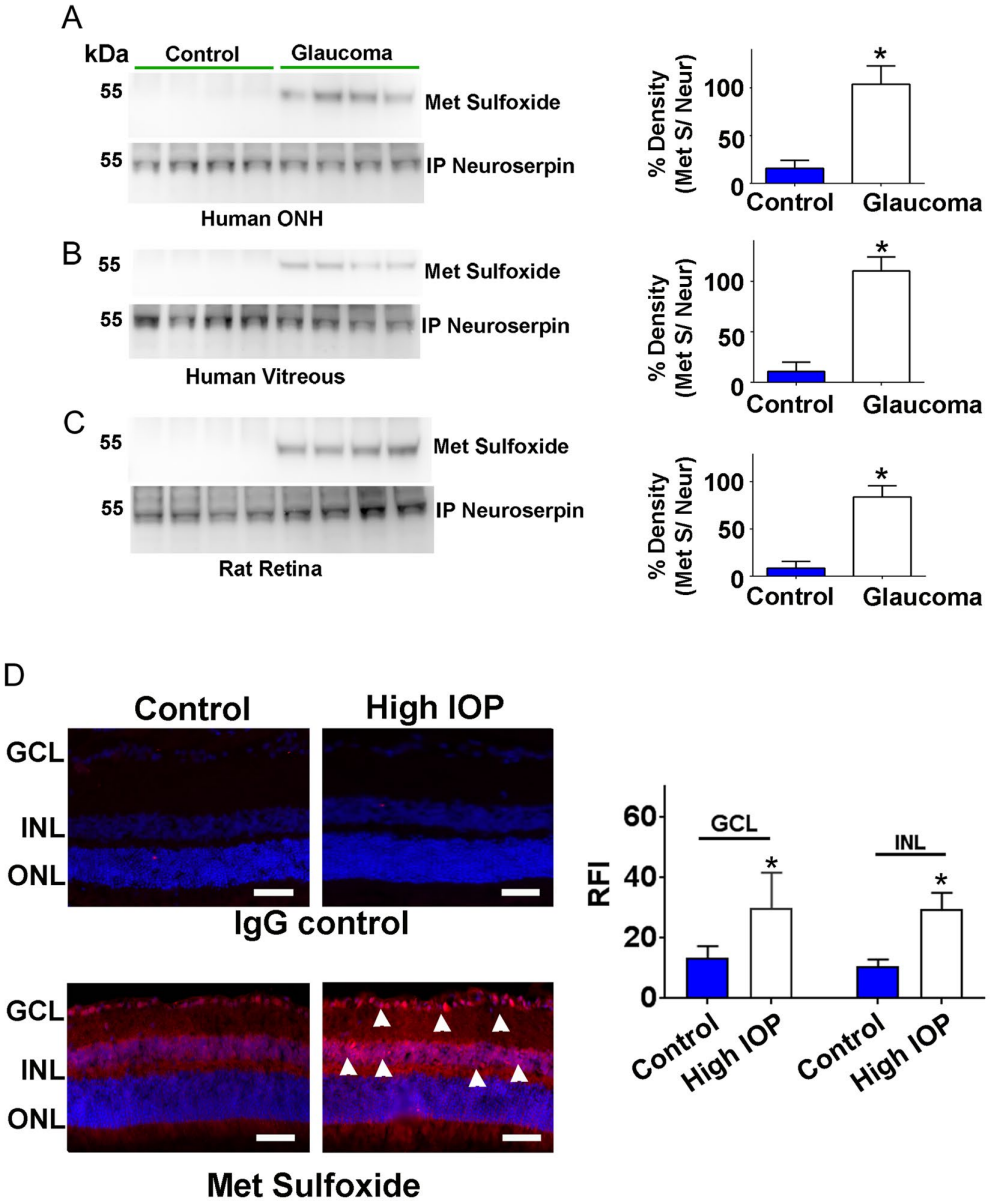
Neuroserpin was immunoprecipitated from control and glaucomatous (**A**) human retinas (**B**) vitreous and from (**C**) the rat retinas. The immunoprecipitates were blotted and probed for methionine sulfoxide immunoreactivity. The blots were also probed with anti-neuroserpin antibody in each case. Blots were cropped to show the relevant bands. Relative band intensities were quantified in each case and plotted (n = 4; *p < 0.001). (**D**) Rat retinal sections from control and high IOP induced eyes were immunostained with either IgG control or met sulfoxide antibody (red), DAPI (blue). Arrows (white) highlight the increased met sulfoxide staining in the inner retinal layers of high IOP subjected rats. The average relative fluorescence intensities for met sulfoxide reactivity in sections (n = 4) were quantified in GCL and INL of retinas using ImageJ, compared using students t-test and plotted with standard deviation and error bars (Scale 50 µm, *p < 0.04).

**Figure 8:**
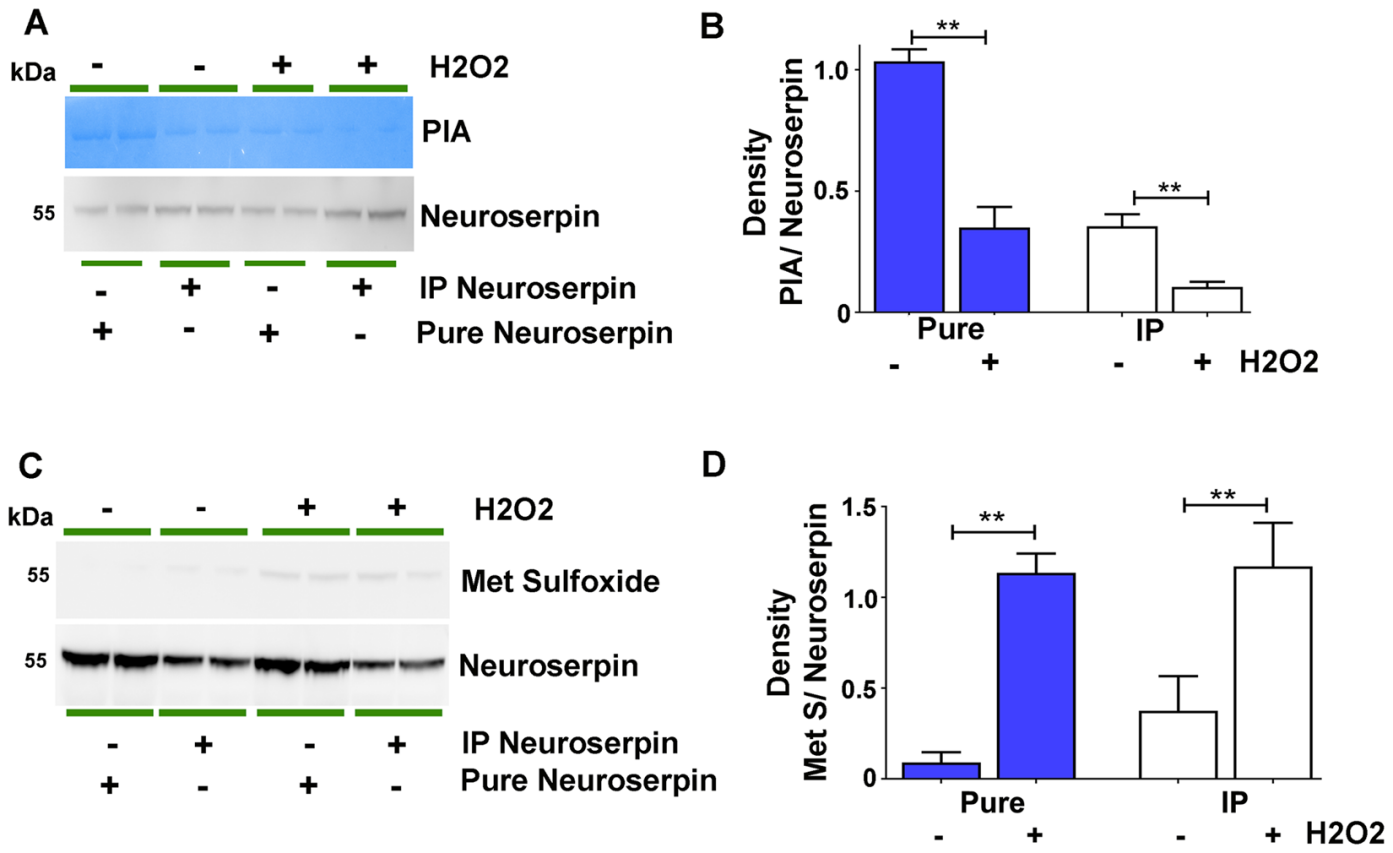
Purified neuroserpin (5 µg) and immunoprecipitated neuroserpin from the normal human retinas (200 µg protein) with or without the H2O2 treatment (10 µM, 1 hr) were subjected to (**A**) gelatin zymography for plasmin inhibitory activity and (**C**) immunoblots probed for met sulfoxide reactivity. (**B**) (**D**) Changes in plasmin inhibitory activity and met sulfoxide reactivity were compared with total neuroserpin blotted in each case and the densitometric data quantified and plotted (**p < 0.01).

Neuroserpin has an exposed methionine residue on its reactive site loop like many other members of the serpin superfamily^26, 27^. The presence of about 20 other methionines in its amino acid sequence along with a critical met residue at reactive site makes the inhibitor sensitive to oxidative stress induced modification to met sulfoxide^28^. Accordingly, we sought to investigate whether neuroserpin exhibits potential changes in met sulfoxide reactivity under glaucoma conditions, which could be the reason underlying diminished neuroserpin inhibitory activity. Neuroserpin was immunoprecipitated from the human ONH and vitreous tissues, subjected to WB, assessed for met sulfoxide immunoreactivity and the band intensities quantified. Human glaucoma samples depicted consistently elevated met sulfoxide immunoreactivity indicating oxidation of neuroserpin met residues (p < 0.001) (Fig. 7A,B). Neuroserpin was also immunoprecipitated from the control and high IOP exposed rat retinas. The immunoprecipitates were subjected to WB and probed against met sulfoxide reactivity. Similar to the observations in human samples, rat retinas also exhibited enhanced met sulfoxide reactivity only in glaucoma but not in the control samples (p < 0.001). Together these results suggest that glaucoma induced oxidative modification of the met residues in neuroserpin molecule which may be the basis of neuroserpin inactivation in glaucoma (Fig. 7C). The WB results were also validated using immunohistochemistry in the rat retinal sections exposed to high IOP. Enhanced met sulfoxide fluorescence predominantly localised to the inner retina especially the GCL and INL under glaucoma conditions was observed (Fig. 7D). The densitometric quantification revealed significantly elevated relative fluorescence intensity of met sulfoxide under high IOP conditions (p < 0.04). Met oxidation in neuroserpin molecule and its functional effect on plasmin inhibitory activity were further validated by incubating purified neuroserpin as well as neuroserpin immunoprecipitated from human retina with H2O2. Incubation with H2O2 resulted in significant decrease in plasmin inhibitory activity of the neuroserpin (Fig. 8A,B) (**p < 0.01). The decreased plasmin inhibitory activity of neuroserpin inversely corresponded with enhanced met sulfoxide reactivity of the serpin upon H2O2 treatment (Fig. 8C,D) (**p < 0.01). This study demonstrated enhanced met oxidation in neuroserpin in glaucoma and under the oxidising conditions but does not implicate any particular met residue in the process.

**Figure 9:**
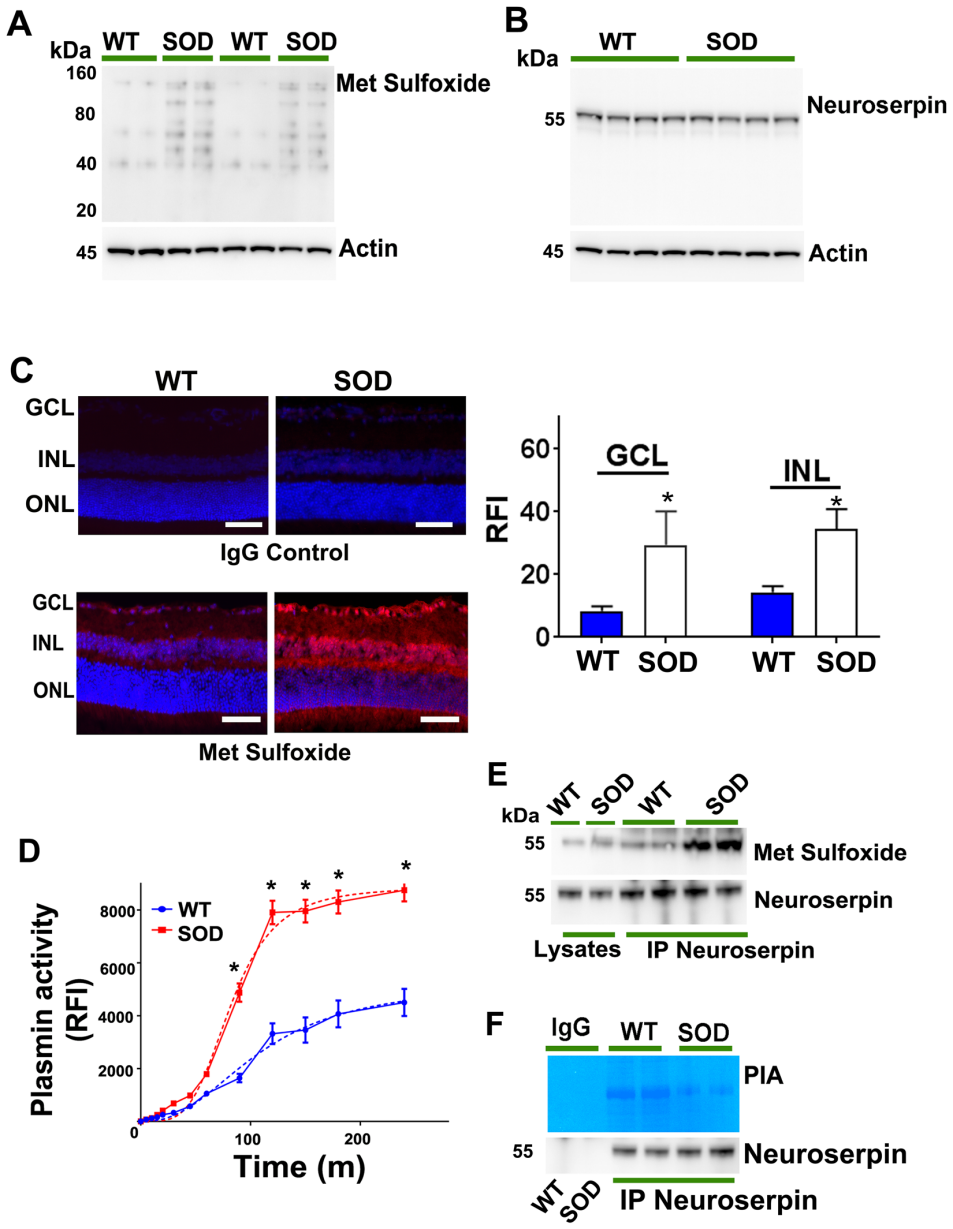
Retinal lysates from WT and SOD mutant mice were immunoblotted and developed for (**A**) methionine sulfoxide and (**B**) neuroserpin reactivity (n = 4). Actin was used as internal control for normalisation in each case. (**C**) Increased met sulfoxide immunofluorescence (red) was observed in the retinal sections from SOD mutant mice and results compared with wild type animals and also with the sections incubated with control IgG antibodies (Dapi- blue). The relative fluorescence intensities for met sulfoxide reactivity in WT and SOD sections were quantified in the GCL and INL using ImageJ and data plotted (Scale 50 µm, n = 4, *p < 0.05). (**D**) Plasmin proteolytic enzymatic assay was carried out (0–300 minutes) from retinal lysates obtained from WT and SOD mutant retinas and plotted (n = 4). Dotted lines represent sigmoidal allosteric regression analysis (*p < 0.05). (**E**) Neuroserpin immunoprecipitates from WT and SOD mutant mice retinas along with retinal lysates were subjected to western blotting for met sulfoxide immunoreactivity. (**F**) Following immunoprecipitation from WT and SOD mutant mice retinal lysates with anti-neuroserpin antibody, immunoprecipitates were subjected to gelatin in-gel zymography for plasmin proteolytic inhibitory activity assay. Non-immune IgGs were used as control for immunoprecipitation.

In order to understand whether met sulfoxide reactivity was associated with increased oxidative stress *in vivo*, we probed the retinal lysates of adult WT and SOD mutant (G93A) mice with met sulfoxide antibody using WB. Significantly increased met sulfoxide staining was observed in SOD mutant mice retinal lysates upon densitometric quantification compared to WT counterparts (Figs 9A and S6A) (p < 0.05). These changes were not associated with any alterations in neuroserpin expression in SOD mutant mice retinas as determined by WB (Fig. 9B). Met sulfoxide antibody staining was also carried out on the retinal sections and revealed increased immunofluorescence in the SOD mutant mice retinas compared to WT counterparts (Fig. 9C). Densitometric quantification showed enhanced met sulfoxide fluorescence particularly in the GCL and INL of retina (p < 0.05). We further evaluated whether plasmin activity in SOD mutant mice retinas showed any alterations corresponding to reduced neuroserpin activity. Time dependent analysis (0–300 minutes) demonstrated significantly elevated plasmin activity in SOD mutant mice retinas (p < 0.05) compared to the WT animals exhibiting a non-linear allosteric curve (R^2^; WT = 0.96; SOD = 0.98) (Fig. 9D). Met oxidation of the neuroserpin molecule in SOD mice was specifically tested by immunoprecipitating neuroserpin from mutant and WT retinas and probing the WB against met sulfoxide antibody. Increased neuroserpin met sulfoxide reactivity was detected in retinal samples from SOD mice compared to WT animals (Figs 9E and S6B) (p < 0.05). Neuroserpin immunoprecipitation and in-gel gelatin zymography from SOD mutant retinas followed by densitometric quantification demonstrated significantly downregulated plasmin inhibitory activity compared to WT mice (Figs 9F and S6C) (p < 0.05). Immunoprecipitation with non-immune IgGs was used as control. These data match with the overall enhanced met sulfoxide reactivity observed in SOD mutant mice retinas. Together, these results supported the premise that oxidative inactivation of neuroserpin in glaucoma corresponded with increased plasmin proteolytic activity in the retina.

### Retinal extracellular matrix protein degradation in glaucoma

**Figure 10:**
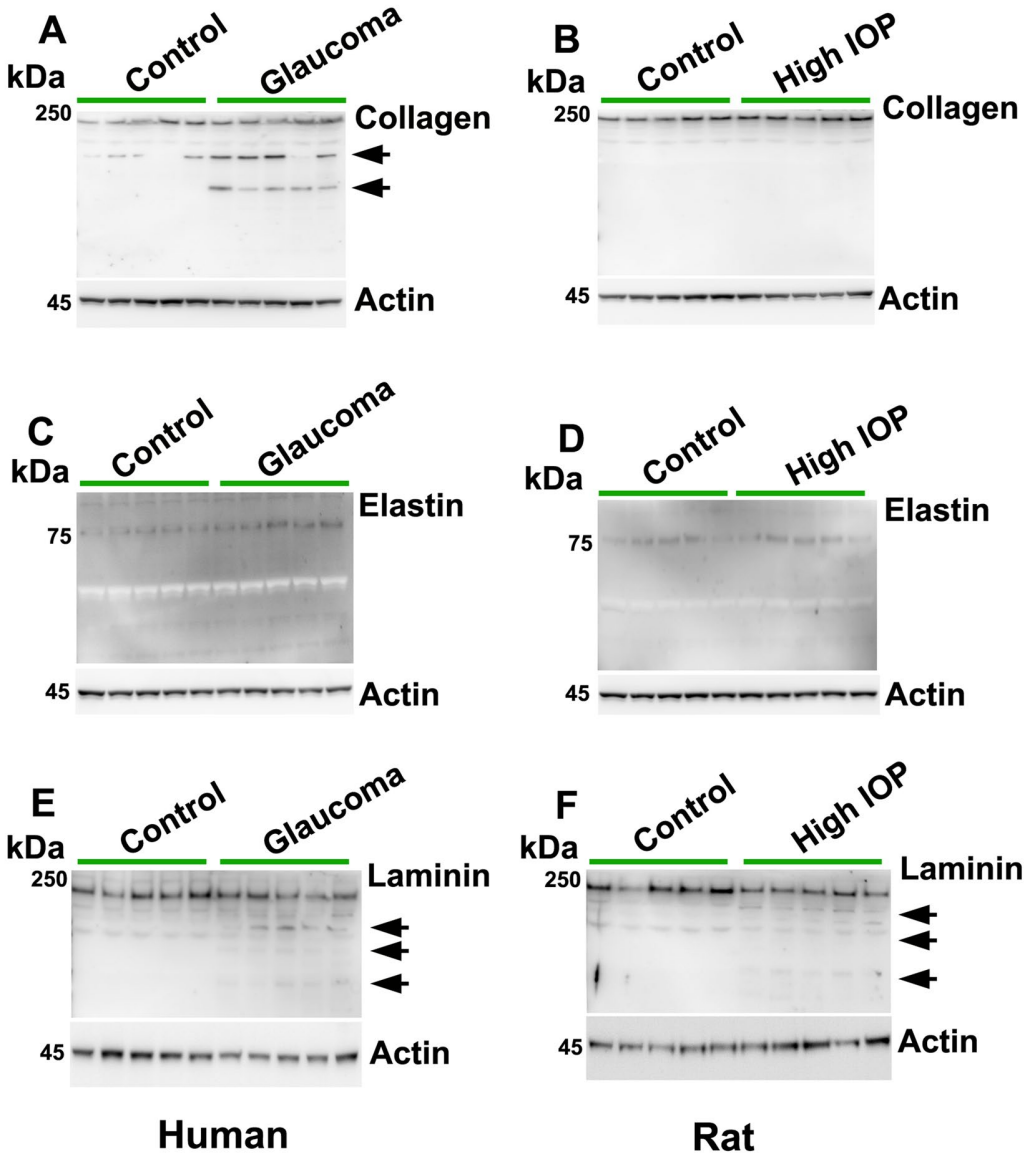
Control and glaucomatous human (n = 10) and rat retinal tissue lysates (n = 5) were immunoblotted and probed for (**A,B**) collagen **(C,D**) Elastin and (**E,F**) laminin immunoreactivity. Arrows indicate degradation products of the ECM proteins in the glaucomatous samples. Actin was used for protein normalisation in each case.

Whether enhanced proteolytic activity of plasmin in glaucoma was associated with changes in ECM degradation was investigated by subjecting the retinal lysates to immunoblotting and probing with antibodies against extracellular proteins including collagen, elastin and laminin. Human retinal tissue analysis from glaucoma subjects using immunoblotting demonstrated increased collagen degradation while no collagen degradation was observed in the rat retinal lysates exposed to chronically increased IOP (Fig. 10A,B). This data suggested differential regulation of ECM protein degradation in rat glaucoma model compared to that of the human glaucoma conditions. No elastin degradation products were detected in either human or rat retinal samples (Fig. 10C,D). Interestingly, laminin degradation products were identifiable in both human and rat retinal tissue WB (Fig. 10E,F). Actin was used as loading control in each case. These results corresponded with the evidence of enhanced plasmin activity and reduced protease inhibitory activity of neuroserpin marked by its enhanced met oxidation under the glaucomatous conditions, although it is acknowledged that this does not necessarily indicate that ECM remodelling in glaucoma was directly caused by plasmin activation or neuroserpin oxidation.

## Discussion

Our results indicated that the retinal and vitreous tissues of human glaucoma subjects and retinas of animal model of chronically increased IOP demonstrated enhanced plasmin amidolytic activity and reduced neuroserpin protease inhibitory activity. These alterations in activity were not accompanied by either plasmin or neuroserpin protein expression or localisation related changes. Glaucoma or high IOP exposure resulted in met oxidation of the neuroserpin molecule resulting in loss of its plasmin inhibitory activity. Increased plasmin activity, enhanced met sulfoxide oxidation of neuroserpin and consequent loss of its protease inhibitory activity in glaucoma retinas was partly recapitulated in SOD G93A transgenic mice that are exposed to high levels of oxidative stress as well as *in vitro* by incubating purified neuroserpin with H2O2.^29^. The observations of enhanced proteolytic activity in retinas were strongly supported by evidence of increased ECM degradation products in the retinas under glaucomatous conditions.

Neuroserpin has previously been shown to bind to plasmin and its activators^30^. Elevated levels of tPA and uPA have been shown to induce RGC loss in kainic acid excitotoxicity model of RGC damage^31^. Similarly, in an animal model of increased IOP, tPA and uPA upregulation was observed to induce RGCs loss and inhibiting their proteolytic activity imparted protection to the RGCs^32^. In accordance with this study, we also observed increased uPA activity in the rat retinas that were exposed to increased IOP and no significant changes were observed in tPA activity at 2 months’ time point^32^. An increase in tPA expression levels was observed in the whole rat retinal lysates. Enhanced tPA expression in the RGCs of increased IOP model has been reported previously^32^. Although, retinal astrocytes have been shown to depict upregulated uPA levels using immunohistochemistry in the increased IOP model^32^, this altered expression was not evident in WB performed using whole retinal lysates. Interestingly, neither tPA nor uPA expression or enzymatic activities were significantly altered in human glaucoma samples indicating that plasmin activation observed in glaucoma pathology could not possibly be attributed directly to tPA or uPA activation. These findings are in agreement with previous studies that show that neuroserpin may function by mechanisms beyond the inhibition of tPA. For example, Wu *et al*. (2010) reported that neuroprotective effects of neuroserpin by plasmin mediated cell death in ischemia were independent from its inhibitory effects on tPA^29^. In the retinal tissue, Gu *et al*. (2015) demonstrated using tPA^-/-^ mice that neuroserpin protected against retinal injury independent of its effects on tPA^23^. Furthermore, we observed that purified neuroserpin, similar to the neuroserpin immunoprecipitated from the retina using specific antibodies showed strong plasmin inhibitory activity (Figs 5 and 8A). Thus, although neuroserpin does have a known inhibitory effect on plasminogen activators particularly tPA, our study indicated that the serpin could also directly affect the plasmin activity in the retina.

Neuroserpin increase subsequent to stroke incidence is clinically associated with improved functional recovery^21^. On the other hand, deficiency of this molecule is demonstrated to aggravate inflammation in the CNS after stroke^11^. We observed neuroserpin plasmin complex under glaucomatous conditions both in the human and in the rat retinas exposed to chronically increased IOP. This suggested that a fraction of neuroserpin mediated neutralisation of plasmin formed a stable complex and this could be explained by suicide inhibition mode of serpins to neutralise proteases. Active neuroserpin and neuroserpin-tPA complexes have previously been reported to be present in cortical cultures and embryonic fibroblasts and internalised in a low density lipoprotein receptor related protein (LRP) mediated process^33^. A neuroserpin: tPA complex band has also previously been identified in brain extracts of mice overexpressing neuroserpin^8^. Neuroserpin plasmin complex formation observed in the present study, indicated that neuroserpin interacted with plasmin *in vivo* and that complex formation was increased under glaucoma conditions. Hastings *et al*. (1997) reported that neuroserpin reacted slowly with plasmin and Osterwalder *et al*. (1998) suggested substrate like activity of neuroserpin; both these studies along with Krueger *et al*. (1997) support that small amount of high molecular weight SDS stable neuroserpin-plasmin complex formation was observed, possibly with a higher stoichiometry ratio^10, 34, 35^. Our study suggested that a pool of the neuroserpin protein could form a relatively more stable complex with the plasmin under glaucoma conditions. While this likely indicates enhanced protease-serpin interactions in the retina in glaucoma, it could potentially also be caused by combination of several other factors such as conformational changes in neuroserpin upon oxidation^36^ leading to a neuroserpin-plasmin complex formation *in vivo* that is better recognised by the immunoprecipitating antibodies or reduced clearance of the plasmin-neuroserpin complex from the retina under glaucoma conditions.

Neuroserpin contains about twenty met residues, which makes the molecule susceptible to oxidative changes as suggested previously^37^. Oxidative stress can modify the met residues including the reactive site loop met residue to met sulfoxide, as shown in the case of other serpins such as alpha-1-proteinase inhibitor and potentially decrease the proteinase inhibitory potential of neuroserpin^26, 27^. Epidemiological evidence indicates that glaucoma pathology is associated with increased oxidative damage^38, 39^. Accordingly, we observed increased met sulfoxide reactivity of neuroserpin and its reduced inhibitory activity which may be attributed to chronically increased oxidative stress under glaucoma conditions, making neuroserpin labile to oxidative inactivation. This study however does not implicate oxidation of reactive site loop met or any other specific met residue in the molecule. Loss of this protease inhibition mechanism of neuroserpin could impair plasmin regulation by alleviating its inhibition and may exacerbate proteolytic degradation of ECM proteins.

The involvement of oxidative stress in mediating oxidative inactivation of neuroserpin was confirmed by incubating purified neuroserpin with H2O2 *in vitro* as well as in the SOD mutant animals *in vivo* that exhibit increased oxidative stress. SOD catalyses the conversion of O^2-^ radical to H_2_O_2_, which is then removed by catalase and glutathione peroxidase by converting it into to H_2_O and O_2_^40^. Both H_2_O_2_ and O2- can penetrate through the membrane and affect the endoplasmin reticulum (ER) redox environment^41^. ER in particular, has an oxidisable environment due to low GSH: GSSG ratio that makes the ER proteins more sensitive to oxidation^42^. Thus, SOD may indirectly regulate the oxidation of neuroserpin in ER through its ROS scavenging actions. SOD is abundantly present in the retina and is shown to protect the retina against ischemia induced injury^43^. Previous studies have reported that SOD1^-/-^ mice depicted progressive loss of retinal electrophysiological responses, retinal thinning and degenerated mitochondria^44, 45^. SOD1 null animals also revealed cumulative oxidative damage to various cells of the retina, drusen formation and retinal pigment epithelium (RPE) degeneration^46^. SOD overexpression was also shown to accelerate RGC degeneration in optic nerve crush mouse model and this was attributed to accelerated oxidative damage caused by H_2_O_2_ accumulation^40^. The present study demonstrated that SOD mutant mice showed increased oxidative inactivation of neuroserpin as evidenced by significantly higher met sulfoxide reactivity of the molecule. These experiments also supported the argument that cytoplasmic scavenger SOD1 isoform specifically plays an important role in protecting neuroserpin from oxidative inactivation as other isoforms of SOD (SOD2 and 3) were still present in these mice. Overall, our findings in glaucoma and oxidative stress conditions are in agreement with previous study and showed that neuroserpin activity was not completely lost in response to oxidative environment, although it was significantly reduced^36^.

In addition to oxidative inactivation, a fraction of the neuroserpin may be sequestered by interacting with amyloid β aggregates. Neuroserpin was shown to interact with amyloid β aggregates in the brain and retina. The presence of protease inhibitor was implicated in making Aβ aggregates more resistant to proteolytic digestion and limit the ability of the plasmin to mediate Aβ clearance^47–49^ Accordingly, neuroserpin deficient mice demonstrated increased clearance of Aβ and reduced Aβ deposition in brain^50^. We recently reported Aβ accumulation in human retinas from glaucoma subjects and in the retinas of animal models of glaucoma^51, 52^.

There are several reports in the literature indicating that plasmin activation might play an important role in RGC death^31, 32, 53^. Sustained plasmin activation may exacerbate ECM remodelling and promote ONH excavation. Our study corroborated previous reports suggesting enhanced proteolysis and ECM degradation at the ONH and also identified collagen and laminin degradation products in the human retina under glaucoma conditions^54^. The ECM is composed of loose basement membranes, isolated bundles of collagen fibers, and other proteins such as laminin and elastin that resist structural deformation of the tissue^55^. Laminin, collagen and elastin are important ECM components believed to facilitate cell attachment and play a role in cellular signalling. RGC degeneration in glaucoma has been shown to correlate with IOP-induced changes in specific ECM constituents including laminin and collagen degradation suggesting that abnormal ECM remodelling may be associated with RGC death^3^. Decreased collagen content was also observed in monkey and human ONH in glaucoma^56^. While notable collagen degradation was observed in human glaucoma samples, laminin degradation products were identified in both human glaucoma samples and in rat glaucoma tissues. These differences could be explained based on the fact that while human glaucoma is complex condition that involves retinal degenerative changes sometimes independent of the IOP, the rat model only mimics one component of the glaucoma which is increased IOP. The collagen specific differences between human samples and rat model could also possibly be attributed to species specific differences. ECM remodelling could lead to disrupted cell-cell interactions and induce RGC death. Marked changes in the composition and organization of the ECM of the lamina cribrosa in glaucoma have previously been detected^57, 58^. Alterations in elastin fibres and ECM changes were shown to coincide with visual field defects and ONH reorganisation in glaucoma^55^. We hypothesise that persistent ECM remodelling may potentially limit the elasticity and compressibility of the ONH and lamina cribrosa in glaucoma which in turn could further exacerbate the pressure induced mechanical effects of stress and strain and make the retina more susceptible to neurodegenerative changes. These ECM alterations may gradually affect the molecular architecture and resilience of the optic disc necessary to support RGC axons. In addition, there is evidence to suggest remodelling of the inner limiting membrane which is one of the three basement membranes in the retina, and closely associated with the nerve fibre layer facing vitreous. IOP elevation in the long term, might thus exacerbate the remodelling of ECM and induce damage of ONH and subsequently to ganglion cell body, *vice versa* or by simultaneous injury to both ONH and ganglion cell bodies. This study highlighted that ECM degradation is observed in both human and experimental glaucoma and this associated with neuroserpin oxidation and plasmin activation. Enhanced ECM degradation does not directly mean that it is caused by increased met oxidation in the neuroserpin molecule and subsequent increase in plasmin activity. This is because plasmin also plays an important role in activating other matrix metalloproteinases which may then affect ECM degradation^59, 60^. Future phenotype rescue experiments can be carried out in mice models by over-expressing oxidatively resistant form of neuroserpin in the specific retinal neurons. Exposing these animals to experimental glaucoma will highlight the contribution of neuroserpin oxidation in mediating glaucomatous damage to the retina.

In summary, our study demonstrates that glaucoma involves activation of the plasmin proteolytic system and loss of neuroserpin activity which in turn correlates with enhanced degradation of ECM components in the ONH region. This study highlights the potential role of activating and reinforcing the anti-oxidant pathways to help combat glaucoma induced degenerative changes in the retina. The relevance of neuroserpin oxidation and its interactions with plasmin in other disease conditions such as Alzheimer’s disease is an important future direction that needs to be investigated. Based on our findings in glaucoma and by analogy with other neurodegenerative disease conditions, neuroserpin may be a critical regulatory molecule involved in fine-tuning the plasmin proteolytic system and its downstream effects.

## Materials and Methods

### Chemicals

Anti-Neuroserpin (Santacruz SC32947 and SC48360), anti-plasmin (Santacruz 15036) and anti-methionine sulfoxide (Novus Biologicals NBPI06707SS) antibodies were used for probing western blots and immunohistochemistry of retinal sections. β-actin (Sigma, AC-40), anti-collagen (Abcam ab6586), anti-elastin (Abcam Ab23747) and anti-laminin (Abcam ab11575) antibodies were used for WB. Plasmin was from Sigma, Missouri, USA and recombinant neuroserpin protein from abcam. Plasmin enzyme activity was measured using 96-well microplate format kits from AnaSpec (Sensolyte Assay kit, AnaSpec, Inc., CA AS-72125). Fluorescent polystyrene microbeads were obtained from Invitrogen (FluoSpheres; Invitrogen, Carlsbad, CA) and dimethyl pimelimidate (DMP) was from Sigma, St. Louis, USA. Tissue type plasminogen activator (tPA) (ab108905) and urokinase type plasminogen activator (uPA) (ab108915) activity assay kits were obtained from Abcam. All other analytical grade reagents were from Sigma, USA.

### Human samples

Freshly frozen human cadaver eyes samples were obtained from individuals who consented to the use of their tissues for research purposes. Eyes were obtained from glaucoma (n = 12) and control (n = 12) from the Sydney Eye Bank, Australia. Research was carried out in accordance with the principles outlined in the declaration of Helsinki. The no of samples (n) indicates independent experiments with tissues from different individuals. Ethics approval was obtained from the Macquarie University Human Research Ethics committee. Mean age of tissue donors was 74 years (67 to 82 years; SD 7.4 years). Patient history was obtained from the donors’ medical records.

### Animals

Animal experiments were performed in accordance with the Australian code of practice for the care and use of animals for scientific purposes and as per the ARVO guidelines for the use of animals in ophthalmic and vision research. SOD (G93A) mutant and wild-type (WT) mice were obtained from University of Wollongong, NSW, Australia (https://www.jax.org/strain/002726) and were approved by the Macquarie University, Australia Animal Ethics Committee. The no of samples (n) indicated in each figure represents tissues from different animals. All animals were maintained in the animal house in cyclic light (12 h on; 12 h off; ~300 lx), at 21 ± 2 °C and with *ad libitum* access to water and food. Mice were sacrificed by asphyxiation with CO_2_ followed by cervical dislocation and eyes harvested.

### Western blotting, zymography and enzyme assays

Human vitreous and retinas were carefully harvested from the donor eyes under a surgical microscope. The ONH region was removed from the isolated retinas under the microscope. For the rat and mice eyes, the retinas were carefully extracted using winkling technique. The samples were lysed in lysis buffer (20 mM HEPES, pH 7.4 containing 1% Triton X-100), 30 µg of protein loaded and resolved by SDS-PAGE and transferred to PVDF membranes. The blots were washed with TTBS (20 mM Tris–HCl [pH 7.4], 100 mM NaCl, and 0.1% Tween 20) and blocked with 5% nonfat milk powder in TTBS for 1 h at room temperature. Blots were then incubated with anti-plasmin (1:1000), anti-neuroserpin (1:1000), anti-actin (1:5000), anti-collagen (1:1000), anti-elastin (1:1000) or anti-laminin (1:1000) at 4 °C overnight. After primary antibodies, blots were incubated with horseradish peroxidase (HRP)-linked secondary antibodies and after thorough washing, signal was detected using Supersignal West Pico Chemiluminescent substrate (Pierce). Plasmin, tPA and uPA amidolytic activities were measured using 96-well microplate assay kits as per manufacturer’s instructions. Protease inhibitory assay of neuroserpin was carried out by gelatin embedded PAGE zymography. Briefly, immunoprecipitated protein was analyzed by zymography on precast 10% polyacrylamide gels containing 1% (w/v) gelatin (Life Technologies, Grand Island, NY, USA). After electrophoresis the gels were incubated at 37 °C in 0.1 M sodium phosphate buffer, pH 7.4, containing plasmin (Sigma) for 1 h by protocol described previously^26, 61^. Dark blue bands against a light background after staining with Coomassie solution indicated neuroserpin. Bands were detected using an automated luminescent image analyser (ImageQuant LAS 4000). Image J programme (NIH, USA) was used to quantify the WB band intensities.

### Immunoprecipitation

Immunoprecipitation was carried out as described previously^62^. Retinal, vitreous and ONH tissues were lysed in the lysis buffer. Insoluble material was removed by centrifugation at 17,000 g for 20 min at 4 °C, and the solubilized proteins (200 µg) were incubated with 40 μl of protein A sepharose for 1 h at 4 °C with continuous mixing. The supernatants were incubated with primary antibodies or normal immunoglobulins overnight at 4 °C and afterwards with 40 μl of protein A-Sepharose for 2 h at 4 °C. This was followed with centrifugation at 17,000 g for 1 min at 4 °C, antigen-antibody complexes were washed thrice with ice-cold wash buffer [50 mM HEPES (pH 7.4) 118 mM sod. chloride, 100 mM sod. flouride, 2 mM NaVO3, 0.1% SDS and 1% Triton X-100], boiled with sample buffer, centrifuged (17000 g × 1 min) and supernatants loaded on to the gel. All immunoprecipitations and gels were carried out without β-mercaptoethanol under non-reducing conditions to minimise heavy and light chains bands originating from antibody breakdown^63, 64^. For protein-protein interaction analysis using co-immunoprecipitation and mass spectrometry the antibodies were cross-linked with sepharose beads (DMP) to eliminate interference from the immunoprecipitating antibodies^65, 66^. The bound proteins were gently eluted with 0.2 M Gly–HCl, pH 2.5 containing 0.5 M NaCl. The immunoprecipitates were subjected to WB with antibodies indicated in the respective figures.

### Immunohistochemistry

Following transcardial perfusion with 4% paraformaldehyde (PFA), eye globes were harvested and subjected to 4% PFA incubation for 1 hr followed by overnight incubation in 30% sucrose solution, cryosections (15 μm) were then permeabilized with cold ethanol and subjected to incubation with primary antibodies (1:100) overnight at 4 °C^67^. Following this sections were incubated with Alexa-Fluor 488 or Cy3 secondary antibodies (1:400 in Tris phosphate buffered saline) for 1 hour in the dark and mounted on slides with medium containing DAPI (Vectashield). The pictures were captured using a microscope (Carl Zeiss) as described previosuly^68^. For quantification of immunofluorescence changes, areas of the retinal layer (GCL and INL) were selected followed by measurement of fluorescent intensity in equal areas using ImageJ software (NIH, USA). The relative fluorescent intensity for various antibodies was compared between the two groups using students t-test. Data was presented as mean ± SD with error bars.

### Intra-ocular injections and pressure measurements

A chronic model of experimental glaucoma was generated in rats by a producing an increase of IOP using microbead injections (Fluospheres, 10 μm) as described previously^2^. Weekly injections into the anterior chamber of eye were made for a sustained increase in IOP for 2 months following which the tissues were harvested. Microbeads were administered using a 25-μL syringe connected to a disposable 33-gauge needle (TSK Laboratory, Japan). All injections were performed under an operating microscope (OPMI Vario S88, Carl Zeiss, Germany). Care was taken to avoid needle contact with the iris or lens and minimise damage. The needle was inserted beneath the corneal surface and 5 μL of beads were injected. Rodents were anesthetized using ketamine (75 mg/kg) and medetomidine (0.5 mg/kg) intraperitoneally^69^, and anaesthesia reversed using atipamazole (0.75 mg/kg injection. 0.3% Ciprofloxacin drops (0.3%) (Ciloxan; Alcon Laboratories, Australia) and 0.1% dexamethasone eye drops (Maxidex, Alcon Laboratories) were instilled in the eyes. Lacrilube; (Allergan) was applied to cornea to protect the rodent eyes. IOP was regularly measured with the help of rebound tonometer (Icare Tonovet, Finland). Three consecutive pressure readings were recorded from the eyes and their average taken to determine the intraocular pressure.

### Trypsin in-gel digestion

SDS PAGE lanes were excised (corresponding to each biological replicates) and cut into smaller pieces. Gel pieces were destained using 100 mM NH_4_HCO_3_, followed by two times using 200 µl of ACN (50%)/ 100 mM NH_4_HCO_3_ (50%) for 10 min, and finally with 100% ACN. After drying samples were alkylated using 50 µl of 50 mM iodoacetamide/NH_4_HCO_3_ (50 mM) for one hour. Samples were washed with 100 mM NH_4_HCO_3_, 200 µl of ACN (50%)/ 100 mM NH_4_HCO_3_ (50%) for 10 min, dehydrated with 100% ACN and then dried. 20 µl of trypsin (in 50 mM NH4HCO3) was used for sample digestion overnight at 37 °C. The digested peptide products were extracted 2 times with 30 µl ACN (50%)/ formic acid (2%), dried, and resuspended in 10 µl of 2% formic acid for further analysis.

### Nanoflow LC-MS/MS

Two samples per replicate were analysed on a Q-Exactive Orbitrap, Thermo Scientific mass spectrometer coupled to an EASY-nLC1000^70^. C18 reverse-phase chromatographic separation was carried out on a column with 75 μm internal diameter × 100 mm, HALO column with 160 Å pore size. A linear gradient of 1–30% (99.9% acetonitrile containing 0.1% FA) was run over 170 minutes. Mass spectra was acquired over the m/z range 350 to 1850 at 35,000 resolution and an automatic gain control target value of 1 × 10^6^ ions. The ten most abundant ions were selected for higher energy collisional dissociation at isolation width of 3.0 m/z. The nanospray ionisation source was operated using a spray voltage of 2.2 kV, spray current of 1.3 µA, S–lens RF level 50.0, and capillary temperature set at 250 °C.

### Data processing and protein identification

The files were generated using Xcalibur programme (Thermo Scientific) and Proteome Discoverer v1.4 and MASCOT (Matrix Science, UK) was used to further process the data. The spectra were compared against the human proteome database (NCBI). A limit of 0.1 Da was applied for MS/MS tolerance and MS tolerance was set to 10 ppm. False discovery rates were minimised and trypsin contaminants eliminated by comparison against reverse sequence database. Only peptides with a score >15 were included with a minimum threshold of Mascot significance set at p = 0.05. Protein abundance was analysed based on normalized spectral abundance factors (NSAF) results as detailed previously^71, 72^.

### Statistical analysis

Changes in protein expression levels in SDS-PAGE and immunoblots, differences in enzymatic activity, expression changes in retinal sections and intraocular pressure differences were compared between the experimental and control groups. Data were analysed and plotted using GraphPad Prism software (v 6.0) (GraphPad Software, San Diego, CA). The values are presented along with error bars as mean ± standard deviation for given sample sizes (n) and compared by Student’s *t* test for unpaired data. The significance was set at *p* < 0.05.

## Electronic supplementary material

Supplementary data
