# Supplementary material for: Glaucoma is associated with plasmin proteolytic activation mediated through oxidative inactivation of neuroserpin
Source: Sci Rep. 2017 Aug 21;7:8412. doi: 10.1038/s41598-017-08688-2 (PMC5566433; doi:10.1038/s41598-017-08688-2)
Supplement: Supplementary file 1 — Supplementary data [file 41598_2017_8688_MOESM1_ESM.pdf]

Glaucoma is associated with plasmin proteolytic activation mediated through oxidative inactivation of neuroserpin

Vivek Gupta[1*], Mehdi Mirzaei[2], Veer Bala Gupta[3], Nitin Chitranshi[1], Yogita Dheer[1], Roshana VanderWall[1], Mojdeh Abbasi[1], Yuyi You[4], Roger Chung[1], Stuart Graham[1,4]

[1]Faculty of Medicine and Health Sciences, Macquarie University, Sydney, Australia.

[2]Department of Chemistry and Biomolecular Sciences, Macquarie University, Sydney, Australia.

[3]School of Medical Sciences, Edith Cowan University, Perth, Australia.

[4]Save Sight Institute, Sydney University, Sydney, Australia.

*Corresponding author:

Faculty of Medicine and Health Sciences, 75, Talavera Road,

Macquarie University, Sydney, NSW 2109, Australia.

Tel.: +61 298502760.

E-mail address: vivek.gupta@mq.edu.au

Legends to supplementary figures

S1: Microbeads were injected weekly into the rat eyes to induce increased IOP (2 months). Microbead injected eyes demonstrated a sustained increase in IOP compared to the control eyes (*$p<0.05$) as measured by I-care tonometer.

S2: Multiple alignment of human, mouse and rat neuroserpin amino acid sequence indicating that the protein primary structure is highly conserved (red) amongst the three species. Reactive site Arg-Met bond of the serpin is highlighted in the box (cyan).

S3: Western blot showing tissue plasminogen activator (tPA) expression in the **A.** human and **B.** rat retina. The relative intensities of bands in WB were quantified and plotted. Actin was used as internal loading control. (n=4 each; *$p<0.05$). **C.** Time dependent tPA enzymatic assay was carried out (0-8 hrs) from human and rat retinal lysates from control and glaucoma samples (n=5 each) and data plotted. Dotted lines represent allosteric sigmoidal curve regression analysis.

S4: Western blot showing tissue urokinase-type plasminogen activator (uPA) expression in the **A.** human and **B.** rat retina. The relative intensities of bands in WB were quantified and plotted. Actin was used as internal loading control (n=3 each). **C.** Time dependent uPA enzymatic assay was carried out (0-8 hrs) from human and rat retinal lysates from control and glaucoma samples (n=5 each, *$p<0.05$) and data plotted. Dotted lines represent allosteric sigmoidal curve regression analysis.

S5: Plasminogen peptides identified following neuroserpin immunoprecipitation and subjecting the trypsin digest of eluted proteins to mass spectrometric analysis.

S6: The relative intensities of bands in WB from WT and SOD mice (figure 9) were quantified and plotted. **A.** quantification of relative lane intensities (figure 9A) (*p<0.05). **B.** quantification of relative band intensities (figure 9E) (*p<0.05). **C.** quantification of relative band intensities (figure 9F) (*p<0.05).

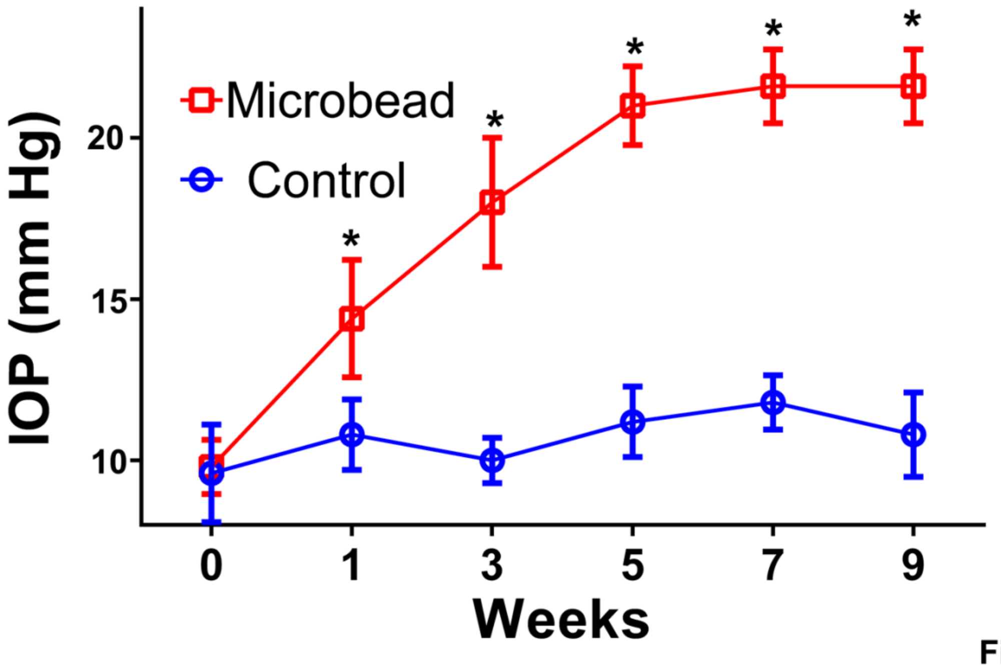

Fig. S1

```
            1        10        20        30        40        50        60        70        80        90        100       110       120       130
            |--------+---------+---------+---------+---------+---------+---------+---------+---------+---------+---------+---------+---------|
NEUS_HUMAN  MAFLGLFSLLVLQSMATGATFPEEAIADLSVNMYNRLRATGEDENILFSPLSIALAMGMMELGAQGSTQKEIRHSMGYDSLKNGEEFSFLKEFSNMVTAKESQYVMKIANSLFVQNGFHVNEEFLQMMKK
NEUS_MOUSE  MTYLELLALLALQSVVTGATFPDETITEWSVNMYNHLRGTGEDENILFSPLSIALAMGMMELGAQGSTRKEIRHSMGYEGLKGGEEFSFLRDFSNMASAEENQYVMKLANSLFVQNGFHVNEEFLQMLKM
  NEUS_RAT  MAYLGLLSLVALQSLVTGAAFPDETIAEWSVNVYNHLRATGEDENILFSPLSIALAMGVMELGAQGSTLKEIRHSMGYESLKSGEEFSFLRDFSSMVSAEEGQYVMKIANSLFVQNGFHINEEFLQMMKM
 Consensus  Ma%LgLlsLlaLQS.vTGAtFP#EtIa#wSVNmYNhLRaTGEDENILFSPLSIALAMGmMELGAQGST.KEIRHSMGY#sLK.GEEFSFLr#FSnMvsAeE.QYVMKiANSLFVQNGFH!NEEFLQM$Km

            131      140       150       160       170       180       190       200       210       220       230       240       250       260
            |--------+---------+---------+---------+---------+---------+---------+---------+---------+---------+---------+---------+---------|
NEUS_HUMAN  YFNAAVNHVDFSQNVAVANYINKWVENNTNNLVKDLVSPRDFDAATYLALINAVYFKGNWKSQFRPENTRTFSFTKDDESEVQIPMMYQQGEFYYGEFSDGSNEAGGIYQVLEIPYEGDEISMMLVLSRQ
NEUS_MOUSE  YFNAEVNHVDFSQNVAVANSINKWVENYTNSLLKDLVSPEDFDGVTNLALINAVYFKGNWKSQFRPENTRTFSFTKDDESEVQIPMMYQQGEFYYGEFSDGSNEAGGIYQVLEIPYEGDEISMMLALSRQ
  NEUS_RAT  YFNAEVNHVDFSENVAVANYINKWVENYTNSLLKDLVSPGDFDAVTHLALINAVYFKGNWKSQFRPENTRTFSFTKDDESEVQIPMMYQQGEFYYGEFSDGSNEAGGIYQVLEIPYEGDEISMMLVLSRQ
 Consensus  YFNAeVNHVDFS#NVAVANyINKWVENyTNsLlKDLVSP.DFDavT.LALINAVYFKGNWKSQFRPENTRTFSFTKDDESEVQIPMMYQQGEFYYGEFSDGSNEAGGIYQVLEIPYEGDEISMMLvLSRQ

            261      270       280       290       300       310       320       330       340       350       360       370       380       390
            |--------+---------+---------+---------+---------+---------+---------+---------+---------+---------+---------+---------+---------|
NEUS_HUMAN  EVPLATLEPLVKAQLVEEWANSVKKQKVEVYLPRFTVEQEIDLKDVLKALGITEIFIKDANLTGLSDNKEIFLSKAIHKSFLEVNEEGSEAAAVSGMIAISRMAVLYPQVIVDHPFFFLIRNRRTGTILF
NEUS_MOUSE  EVPLATLEPLLKAQLIEEWANSVKKQKVEVYLPRFTVEQEIDLKDILKALGVTEIFIKDANLTAMSDKKELFLSKAVHKSCIEVNEEGSEAAAASGMIAISRMAVLYPQVIVDHPFLYLIRNRKSGIILF
  NEUS_RAT  EVPLATLEPLLKPQLIEEWANSVKKQKVEVYLPRFTVEQEIDLKDILKALGVTEIFIKDANLTAMSDKKELFLSKAVHKSFIEVNEEGSEAAVASGMIAISRMAVLFPQVIVDHPFLFLIKNRKTGTILF
 Consensus  EVPLATLEPLlKaQL!EEWANSVKKQKVEVYLPRFTVEQEIDLKD!LKALG!TEIFIKDANLTa$SDkKElFLSKA!HKSfiEVNEEGSEAAaaSGMIAISRMAVL%PQVIVDHPFl%LIrNRktGtILF

            391      400       410
            |--------+---------|
NEUS_HUMAN  MGRVMHPETMNTSGHDFEEL
NEUS_MOUSE  MGRVMNPETMNTSGHDFEEL
  NEUS_RAT  MGRVMHPETMNTSGHDFEEL
 Consensus  MGRVMhPETMNTSGHDFEEL
```

Fig.S2

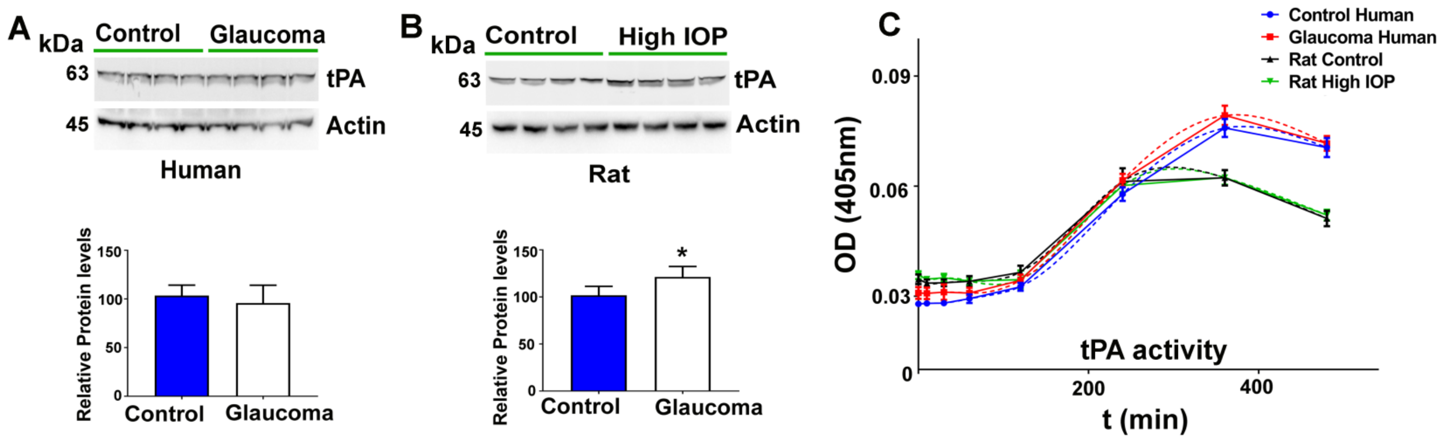
A
kDa
Control
Glaucoma
63
tPA
45
Actin
Human
Relative Protein levels
150
100
50
0
Control
Glaucoma
B
kDa
Control
High IOP
63
tPA
45
Actin
Rat
Relative Protein levels
150
100
50
0
*
Control
Glaucoma
C
Control Human
Glaucoma Human
Rat Control
Rat High IOP
OD (405nm)
0.09
0.06
0.03
0
tPA activity
200
400
t (min)

Fig S3

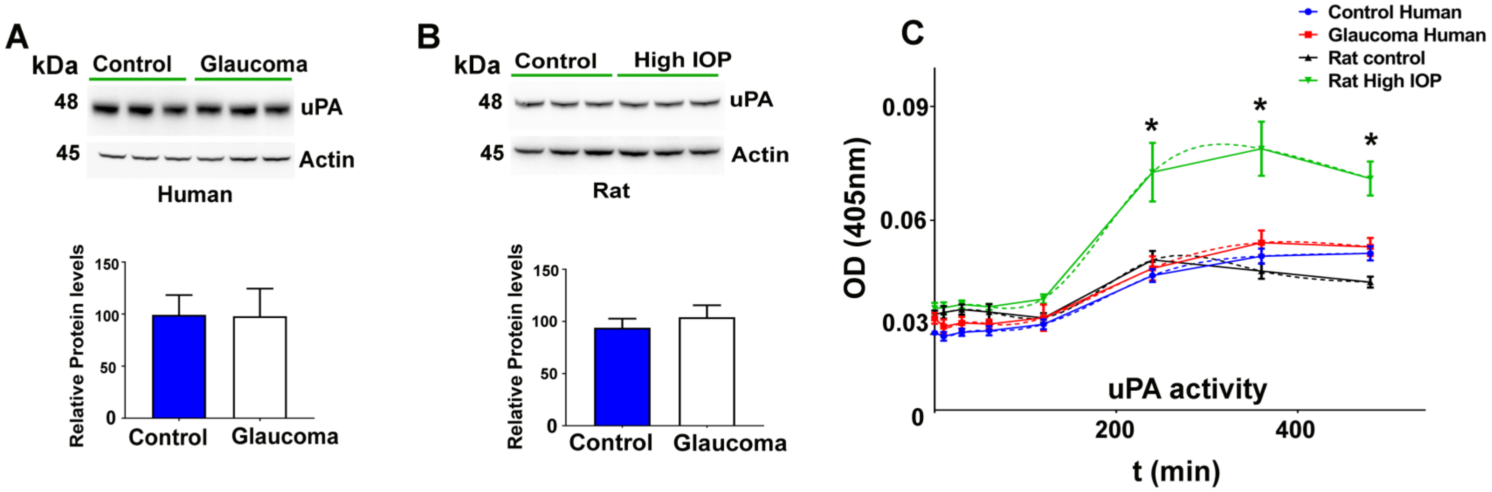
A
kDa
Control
Glaucoma
48
uPA
45
Actin
Human
Relative Protein levels
150
100
50
0
Control
Glaucoma
B
kDa
Control
High IOP
48
uPA
45
Actin
Rat
Relative Protein levels
150
100
50
0
Control
Glaucoma
C
Control Human
Glaucoma Human
Rat control
Rat High IOP
OD (405nm)
0.09
0.06
0.03
0
*
*
*
uPA activity
200
400
t (min)

Fig S4

Plasminogen peptides	
1	KVYLSECK
2	WSSTSPHRPR
3	FSPATHPSEGLEENYCR
4	ELRPWCFTTDPNK
5	RWELCDIPRCTTPPPSSGPTYQCLK
6	NLDENYCRNPDGK
7	TPENFPCK
8	RAPWCHTTNSQVR
9	APWCHTTNSQVR
10	KCQSWSSMTPHR
11	CQSWSSMTPHR
12	TPENYPNAGLTMNYCR
13	HSIFTPETNPR
14	NPDGDVGGPWCYTTNPR
15	LSSPAVITDKVIPACLPSPNYVVADR
16	VIPACLPSPNYVVADR
17	VCNRYEFLNGR
18	FVTWIEGVMR

Fig. S5

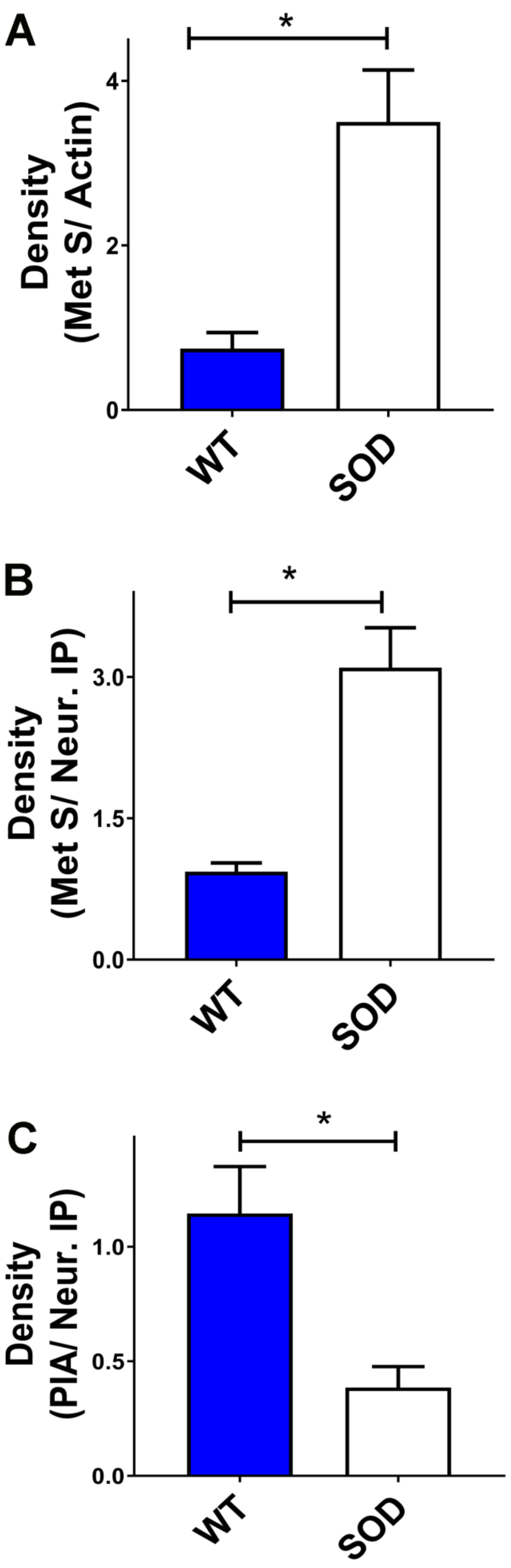
A
*
Density
(Met S/ Actin)
4
2
0
WT
SOD
B
*
Density
(Met S/ Neur. IP)
3.0
1.5
0.0
WT
SOD
C
*
Density
(PIA/ Neur. IP)
1.0
0.5
0.0
WT
SOD

Fig S6
